# Quasi-BIC metasurfaces enable rapid, localized singlet-oxygen generation

**DOI:** 10.1038/s41377-026-02267-9

**Published:** 2026-04-03

**Authors:** Ruilin Long, Laifu Lin, Xinwen Qi, Qiang Liu, Xing Fu

**Affiliations:** 1 Department of Precision Instrument, Tsinghua University, Beijing, 100084 China; 2 State Key Laboratory of Precision Space-Time Information Sensing Technology, Beijing, 100084 China; 3 Key Laboratory of Photonic Control Technology, Ministry of Education, Tsinghua University, Beijing, 100084 China; 4 Department of Chemistry, MOE Key Laboratory of Bioorganic Phosphorus Chemistry and Chemical Biology, Tsinghua University, Beijing, 100084 China

**Keywords:** Nanophotonics and plasmonics, Biophotonics

## Abstract

Bound states in the continuum (BIC) provide a photonic route to concentrate and store electromagnetic energy with minimal radiative leakage. Here, we report Au–TiO_2_ metasurfaces that couple q-BIC optical resonance to interfacial charge transfer, enabling hot-carrier–mediated generation of ^1^O_2_ within subwavelength optical paths. Photonic engineering acts through two cooperative effects: (i) increased optical absorption that boosts hot-carrier generation at the Au/TiO_2_ interface; and (ii) ultralow noble-metal loading that suppresses electron–hole recombination at metal/semiconductor junctions, thereby prolonging carrier lifetimes. These effects jointly yield a synergistic enhancement of ^1^O_2_ production beyond either pathway alone. Under continuous-wave excitation, the metasurfaces reach a local molar-level concentration of ^1^O_2_ within seconds, corresponding to an approximately six-order-of-magnitude increase in local ^1^O_2_ concentration compared with conventional approaches. Tuning structural asymmetry and excitation wavelength enables wavelength- and position-selective cytotoxicity without additional molecular sensitizers. By decoupling strong absorption from nobel-metal usage while extending hot-carrier persistence in a single platform, BIC-engineered metasurfaces offer a general way to achieve efficient photon-to-chemical conversion at solid–liquid interfaces. This approach is of significant value to rapid-acting photodynamic therapy, selective oxidation, and flow microreactors where low dose and geometric precision are critical.

## Introduction

Singlet oxygen (^1^O_2_), the lowest excited electronic state of molecular oxygen^1–4^, serves as one of critical reactive species in photodynamic therapy^5,6^, photocatalytic oxidation^7,8^, and advanced environmental remediation^9^. Conventional ^1^O_2_ generation relies on molecular photosensitizers, such as porphyrins^10^ and xanthene^11^ dyes, that undergo Type II energy transfer upon photoexcitation^12,13^. While these organic systems achieve moderate quantum yields, they suffer from wavelength-nonspecific activation, rapid photobleaching, and poor biocompatibility—factors that necessitate recurrent dosing and limit clinical precision^14–16^.

Recent advances in nanophotonics employ metallic and semiconductor nanostructures as alternative sensitizers^17–20^, yet their ^1^O_2_ yields remain orders of magnitude below therapeutic thresholds due to fundamental limitations in carrier dynamics and photon management^21^. Metallic nanoparticles leverage localized surface plasmon resonances (LSPRs) to concentrate light into subwavelength volumes, generating hot carriers that can drive oxygen activation^22–25^. However, ultrafast electron-hole recombination (<100 fs) in noble metals restricts catalytic turnover rates. Semiconductor nanoparticles such as titanium dioxide (TiO_2_) possess a bandgap that reduces electron-hole recombination to some extent, yet their singlet oxygen (^1^O_2_) quantum yield still remains very low, and ultraviolet excitation is required, which is fundamentally incompatible with biological tissues^26–28^. Hybrid metal-semiconductor architectures can enhance carrier lifetimes through the Schottky barrier, which effectively suppresses hot electron recombination^29–31^. However, they face a challenging material trade-off: increasing the metal content enhances visible-light absorption and boosts the number of activated carriers, but it also introduces additional recombination centers, thereby reducing carrier lifetimes. On the other hand, designs with reduced metal content sacrifice photon absorption efficiency, thereby limiting the number of separated carriers. This trade-off confines practical ^1^O_2_ concentrations to sub-micromolar levels, which is generally insufficient for targeted therapeutic or catalytic applications^17,18,32,33^.

Bound states in the continuum (BIC) represent a unique class of non-radiative electromagnetic modes that coexist with propagating waves in periodic photonic structures^34–38^. Originating from symmetry-protected destructive interference and governed by symmetry- and topology-protected singularities in momentum space, these states exhibit exceptional light confinement capabilities by suppressing energy leakage through radiation channels^39^. When implemented in dielectric metasurfaces, BICs focus optical energy into deeply subwavelength volumes, which sharply increases the interaction between light and matter^40–42^, creating localized electromagnetic hotspots ideal for driving nanoscale photochemical processes^43–46^. This light-trapping mechanism functions independently of material absorption, providing new opportunities to simultaneously enhance carrier generation and optimize carrier separation efficiency in catalytic systems^47^.

**Fig. 1:**
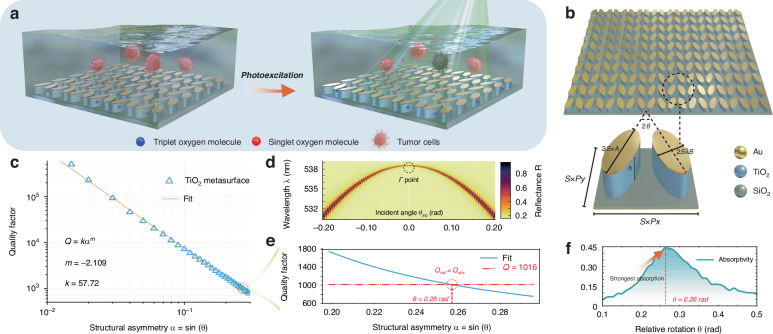
BIC-engineered Au–TiO_2_ metasurface for high-density singlet-oxygen generation. **a** Conceptual illustration of photoexcitation in aqueous media: a q-BIC metasurface concentrates green light to convert triplet oxygen (^3^O_2_) into ^1^O_2_, enabling localized cytotoxicity. **b** Device architecture and unit cell. A square array of TiO_2_ elliptical nanopillars (height *h*) on SiO_2_ is capped with an ultrathin Au layer. An in-plane scaling factor *S* multiplies all lateral dimensions---major/minor diameters *S* × 2*A*, *S* × 2*B* and lattice periods *S* × *P*_*x*_, *S* × *P*_*y*_---while *h* remains fixed. Structural asymmetry is controlled by the relative axis rotation *θ* between neighboring ellipses (asymmetry parameter $\alpha =\sin \theta$). The factor *S* tunes the resonance wavelength without altering *α*. **c** Scaling of the bare TiO_2_ q-BIC quality factor with asymmetry: *Q* = *kα*^*m*^ (fit *m* ≈ −2.109), validating the characteristic *Q* ∝ *α*^−2^ behavior near the symmetry-protected BIC. **d** Angle-resolved reflectance map of the bare TiO_2_ metasurface, showing a narrowband q-BIC dispersion that peaks at the *Γ* point, with its narrowest linewidth at 538 nm (green). **e** Radiative and absorptive quality factors as a function of the structural asymmetry $\alpha =\sin (\theta )$. The radiative quality factor *Q*_rad_ (blue) is obtained by fitting the simulated *Q*-*α* dependence with *Q*_rad_ = *kα*^*m*^, whereas the absorptive quality factor *Q*_abs_ (red dashed line) is calculated from the integrated multilayer system. The intersection *Q*_rad_ = *Q*_abs_ indicates the critical-coupling condition, yielding *θ* ≈ 0.26 rad. **f** Simulated absorptivity of the Au–TiO_2_ metasurface at *λ* = 532 nm as a function of the relative rotation angle *θ*. The absorptivity reaches its maximum at *θ* ≈ 0.26 rad (red dashed line), consistent with the predicted loss-matching (critical-coupling) condition in **e**

We report Au-TiO_2_ metasurfaces that couple q-BIC optical resonance to interfacial charge transfer, enabling rapid, hot-carrier-mediated generation of ^1^O_2_ within a subwavelength optical path and with ultralow noble-metal loading (Fig. 1a). Resonant field confinement boosts absorption while mitigating recombination; the metasurface with the optical thickness of ~100 nm achieves ~45% optical absorption at the wavelength of 532 nm. This enables a local molar-level ^1^O_2_ concentration within seconds, corresponding to an approximately six-order-of-magnitude increase over conventional approaches. Time-correlated single-photon counting of the 1270 nm phosphorescence with spectral filtering validates ^1^O_2_ emission, and complementary frequency-domain analyses together with thermal controls isolate its photochemical origin from photothermal backgrounds. Tuning structural asymmetry and excitation wavelength realizes wavelength- and pixel-level selectivity, enabling rapid, sensitizer-free cytotoxicity. Collectively, these design principles decouple strong absorption from nobel-metal usage and offer a general way to achieve efficient light-to-chemical conversion at solid-liquid interfaces.

## Results

### Mechanism of singlet oxygen generation via BIC-enhanced metasurface

We adopt a critical-coupling strategy built on q-BIC control to maximize interfacial absorption in an ultrathin Au-TiO_2_ metasurface. The unit cell (Fig. 1b) comprises TiO_2_ elliptical nanopillars of height *h* = 100 nm on SiO_2_, capped with a 7 nm Au layer. A weak in-plane symmetry breaking, implemented by a relative rotation *θ* between neighboring ellipses (asymmetry parameter $\alpha =\sin \theta$), lifts the symmetry protection and converts the BIC into a radiative q-BIC. The bare (Au-free) dielectric array exhibits the expected *Q* ∝ *α*^−2^ scaling (Fig. 1c) and a narrowband dispersion with a maximum at the *Γ*-point at 538 nm (Fig. 1d). Introducing the ultrathin Au film opens an absorptive channel. We then tune the asymmetry (rotation angle *θ*) to continuously modulate the radiative leakage and identify the critical-coupling point by the condition *Q*_rad_ ≈ *Q*_abs_^48,49^, which occurs at *θ* ≈ 0.26 rad (Fig. 1e and Supplementary Text 1). At this point, the interfacial absorptance reaches its maximum, yielding ~45% at *λ* = 532 nm within an optical thickness of ~100 nm (Fig. 1f).

**Fig. 2:**
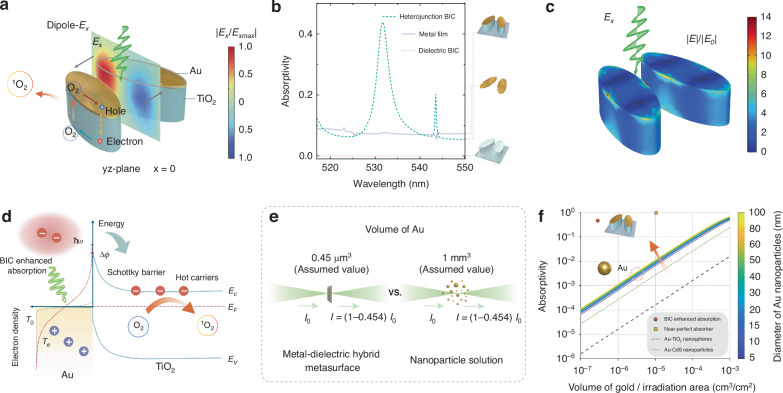
BIC-driven hot-carrier physics in a minimal-metal Au–TiO_2_ heterojunction metasurface. **a** Field localization at the Au/TiO_2_ interface under *x*-polarized illumination. Normalized in-plane field on the *yz*-plane at *x* = 0, $| {E}_{x}/{E}_{x,\max }|$, shows dipolar hot spots that seed interfacial chemistry. **b** Absorptivity spectra for the q-BIC Au–TiO_2_ heterojunction (dashed green), an ultrathin Au film (solid gray), and the bare dielectric q-BIC (dotted red); the heterojunction exhibits a narrow, resonantly enhanced band near the BIC wavelength. **c** Simulated near-field enhancement distribution (∣*E*∣/∣*E*_0_∣) of the Au–TiO_2_ metasurface under *E*_*x*_-polarized illumination at the maximum-absorption condition (corresponding to *θ* ≈ 0.26 rad at *λ* = 532 nm). The map shows the field enhancement on the Au surface and within the underlying TiO_2_ elliptical pillars; the peak enhancement reaches ~14×. **d** Schottky-junction picture: BIC-enhanced absorption raises *T*_*e*_, enabling hot-carrier emission over the barrier Δ*ϕ* from Au (*E*_*F*_) into the TiO_2_ conduction band (*E*_*C*_), thereby driving redox steps that generate ^1^O_2_. **e** Consequence of using less Au: for the same absorbed optical energy, concentration in a smaller metal volume yields higher *T*_*e*_ and, with reduced metal coverage, fewer interfacial recombination pathways---prolonging the catalytic effect. **f** Absorptivity versus Au volume per irradiated area for colloidal nanoparticle references (colored by diameter) and for our BIC metasurface (orange). Same absorption with orders-of-magnitude less Au should be viewed as a functional advantage---higher *T*_*e*_ and suppressed recombination---rather than an economic metric

The optical response directly governs hot-carrier dynamics at the Au/TiO_2_ interface. Near-field simulations confirm an *E*_*x*_-polarized dipolar q-BIC mode (Fig. 2a), and the heterojunction exhibits a narrow absorptivity band pinned to the q-BIC wavelength, which exceeds that of a standalone 7 nm Au film (Fig. 2b). The near-field enhancement at the maximum absorption condition is shown in Fig. 2c, where the optical energy is strongly localized at the rims of the Au nano-ellipses and also penetrates into the underlying TiO_2_ pillars (peak ∣*E*∣/∣*E*_0_∣ ≈ 14). Consistent with this energy localization, two-temperature modeling further predicts rim-localized electron heating within the Au nano-ellipses (Fig. S2). In the Schottky-junction picture (Fig. 2d), BIC-enhanced absorption broadens the electron distribution and enables emission of hot carriers over the barrier Δ*ϕ* ≈ 0.8 eV from Au (*E*_*F*_) into the TiO_2_ conduction band (*E*_*C*_)^50,51^, thereby initiating interfacial redox steps that form ^1^O_2_. By design, BIC photonic engineering localizes the optical field and absorption at the metal rim, enabling the same radiative energy to be absorbed with a substantially smaller amount of metal (Fig. 2e and Supplementary Text 2).

The use of smaller metallic volumes to achieve higher light absorption for ^1^O_2_ generation shows two significant benefits: (i) By enhancing light absorption per unit volume of the metal, the system increases electron activity, which can be quantified by electronic temperature (*T*_*e*_)^52^, thereby improving catalytic efficiency. (ii) Ultralow metal loading mitigates the recombination loss and extends carrier lifetime, thereby enhancing photocatalytic performance.

Our photonically engineered heterostructure outperforms conventional metal-semiconductor hybrids—including near-perfect absorbers^53^, Au-TiO_2_ nanospheres^54^, Au-CdS nanoparticles^55^, and arbitrary-radius nanostructures^56,57^—in decoupling metal loading from optical absorptance (Fig. 2f).

For the equivalent absorbed laser power, the significant reduction in gold volume indicates strong light-matter interactions, leading to a substantial increase in electron temperature (*T*_*e*_). The relationship between *T*_*e*_ and hot-carrier generation efficiency (*J*_*th*_) is expressed as^58^:

1 $${J}_{th}\propto {T}_{e}^{2}\exp (\frac{-q{\phi }_{B}}{{k}_{B}{T}_{e}})$$

where *qϕ*_*B*_ = 0.8 eV and *k*_*B*_ is the Boltzmann constant. When the 532 nm *E*_*x*_-polarized illuminates the metasurface at the focused spot size of 10 μm and the power of 25 mW (*S* = 1.108, *θ* = 0.26 rad), the heterojunction generates a photocurrent of 2.6 pA—a 6.2-fold enhancement over the 0.42 pA produced by conventional Au-TiO_2_ nanoparticles under identical excitation conditions (Supplementary Text 3).

While the photocurrent magnitude differs by a factor of 6.2 between the two approaches, the spatial distribution of current generation shows a fundamentally distinct behavior. In the nanoparticle system, the photocurrent constitutes the sum of microcurrents generated by individual particles dispersed throughout the macroscopic aqueous solution. In contrast, photocurrent generation in the metasurface is highly localized, occurring exclusively within the irradiated region. This spatial confinement enables rapid catalytic conversion of oxygen molecules within micrometer-scale proximity to the metasurface. Combined with the exceptional adsorption capacity of TiO_2_, this configuration produces high ^1^O_2_ concentrations in the immediate vicinity^59^. This q-BIC metasurface establishes synergistic interplay among photonic energy localization, hot electron dynamics, and surface adsorption processes to achieve catalytic enhancement.

### Fabrication and parameter characterization of metasurfaces

**Fig. 3:**
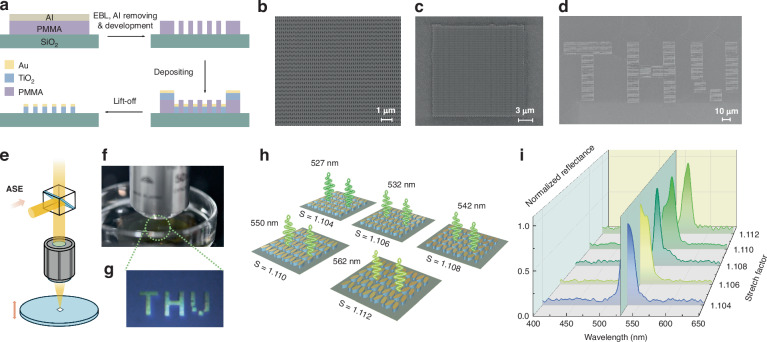
Fabrication, imaging, and spectral multiplexing of Au–TiO_2_ q-BIC metasurfaces. Scanning electron micrographs of representative devices: a uniform array of Au-capped TiO_2_ nano-ellipses (**a**); a stitched metasurface patch (**b**); and a pixelated pattern spelling “THU” (**c**). Scale bars as indicated. **d** Process flow. A PMMA/Al stack on SiO_2_ is patterned by electron-beam lithography (EBL), developed, followed by sequential deposition of TiO_2_ and ultrathin Au; lift-off defines the capped nano-ellipses. **e** Measurement schematic for normal-incidence reflectance under amplified-spontaneous-emission (ASE) illumination and microscope collection. **f** Photograph of the optical setup measuring metasurfaces immersed in aqueous media; **g** bright-field micrograph of the resulting colored “THU” pixels. **h** Spectral multiplexing by geometric scaling. Five metasurfaces with different global stretch factor *S* (uniformly scaling in-plane dimensions while keeping thickness fixed) are designed to target distinct resonances at 527, 532, 542, 550, and 562 nm (*S* = 1.104, 1.106, 1.108, 1.110, 1.112, respectively). **i** Normalized reflectance spectra for the five *S* values, showing a systematic red-shift with increasing *S*, indicating a positive correlation between the resonance wavelength *λ* and *S*. The metasurface of *S* = 1.106 resonates near 532 nm (vertical line), matching the operating wavelength used elsewhere in this work

The q-BIC metasurface is fabricated using micro-nano processing technology (Fig. 3a and Supplementary Text 4). (1) A layer of electron beam resist (polymethyl methacrylate) is coated on a glass substrate, and a thin layer of aluminum is deposited at the bottom of the resist layer to improve conductivity. (2) The resist layer is patterned and subsequently developed using electron beam lithography at 8 C/m^2^, 0.8 nA, and 80 kV. (3) A 100 nm TiO_2_ layer and a 7 nm Au layer are deposited on the imaged resist layer using electron beam evaporation. (4) During the stripping process, the sample is immersed in acetone for 12 hours to remove TiO_2_ and Au in the unexposed areas. After completion, the metasurface is characterized by scanning electron microscopy (Fig. 3b–d).

BIC optical metasurfaces typically exhibit complementary spectral features that include absorption at a specific wavelength and a corresponding reflection peak at nearly the same wavelength. We utilize this phenomenon to precisely characterize the resonant behavior of our metasurface through reflection spectroscopy. The fabricated metasurface is immersed in deuterated water (D_2_O), and a collimated amplified spontaneous emission (ASE) supercontinuum laser is focused on the patterned substrate (Fig. 3e). We optimize the interaction between the beam and the sample by vertically translating the substrate to align the beam waist with the metasurface plane. Since the optimized BIC mode is supported by the *E*_*x*_ polarization, a custom achromatic half-wave plate is used to precisely control the polarization direction perpendicular to the elliptical symmetry axis (transverse polarization), which is crucial for activating the q-BIC mode.

Our custom-built optical microscopy system enables precise positioning of the ASE beam on the metasurface. After optimized focusing, strong green reflectance is visually observed (Fig. 3f), with the fabricated *THU*-shaped pattern (Fig. 3d, g) confirming the designed resonance. To accommodate inevitable nanofabrication errors, we fabricate a sample comprising five metasurfaces with different structural parameters (*S* = 1.104–1.112, Fig. 3h). Hyperspectral imaging is performed using the same microscopy system to capture the reflection spectra of each sample (Fig. 3i). The measured reflection peaks show good agreement with simulations. The moderate broadening likely arises from a combination of (i) fabrication-induced dimensional variations among individual resonators and (ii) the finite angular spectrum introduced by the objective of high numerical aperture. The metasurface with *S* = 1.106 demonstrates optimal resonance at 532 nm and is subsequently selected for ^1^O_2_ generation experiments. The absorptance of the metasurface in aqueous solution is obtained from energy conservation, *A* = 1 − *R* − *T*, where *R* and *T* are the measured specular reflectance and transmittance of the metasurface, respectively. Experimentally, *R* and *T* are determined under 532 nm pulsed-laser excitation using a photodetector (PD) and an oscilloscope by reading the pulse peak amplitude as a linear proxy for optical intensity. For the reflectance measurement, a 50:50 non-polarizing beamsplitter is used and the reflected-signal increment is corrected by a factor of two, while background reflections from the deuterated water surface and the glass vessel are subtracted. For the transmittance measurement, the transmitted signal is recorded directly under the same focusing and collection conditions. The patterned region is compared against an unpatterned area measured in the same optical configuration as a reference. Using this procedure, we obtain *A* ≈ 0.45 at 532 nm, consistent with numerical simulations.

### Steady-state singlet oxygen generation

**Fig. 4:**
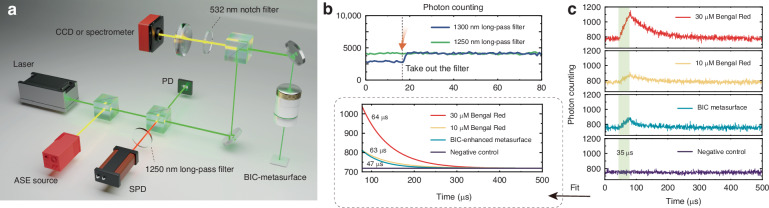
Time-resolved detection of ^1^O_2_ phosphorescence from q-BIC metasurfaces. **a** Optical layout for excitation and near-infrared photon counting. A 532 nm laser (or ASE source) illuminates the BIC metasurface in aqueous media; reflected/elastic light is rejected by a 532 nm notch filter before the CCD/spectrometer, while the NIR arm directs photons through interchangeable long-pass filters (LPF, 1250 or 1300 nm) to a single-photon detector (SPD). A photodiode (PD) provides the timing reference. **b** Filter-removal test for spectral discrimination. Photon-count traces are recorded while sequentially removing the 1250 and 1300 nm LPFs (arrow). Removing the 1250 nm LPF causes no noticeable change in the photon count, whereas removing the 1300 nm LPF produces a pronounced change, confirming that the detected photons have a wavelength near 1270 nm and are attributed to ^1^O_2_ phosphorescence. **c** Top: Time-correlated photon-counting traces for (top to bottom) 30 μM RB, 10 μM RB, BIC-enhanced metasurface, and a negative control. Bottom: representative decay curves and single-exponential fits yield lifetimes of *τ*_RB_ ≈ 64 μs for the RB control and *τ*_MS_ ≈ 47 μs for the BIC-enhanced metasurface, whereas the negative control shows no long-lived component

To investigate the ^1^O_2_ production dynamics of the BIC metasurface in steady-state operation, we perform time-resolved phosphorescence measurements in oxygen-saturated deuterium oxide (D_2_O)^60^. A pulsed 532 nm laser (pulse width of 35 μs, repetition rate of 2 kHz) is used to activate the metasurface, and the ^1^O_2_ phosphorescence signal at 1270 nm is detected using time-correlated single photon counting. Long-pass filters (LPFs) and monochromators are employed to eliminate scattered excitation light, ensuring precise lifetime measurements (Fig. 4a). Unless otherwise stated, all kinetic curves are acquired under a fixed incident optical power (25 mW) at the sample plane (i.e., constant incident photon flux). We validate the phosphorescence wavelength by temporarily removing LPFs with different cutoff wavelengths (1250 and 1300 nm) and monitoring the photon counts; the pronounced change upon removing the 1300 nm LPF, together with the negligible change upon removing the 1250 nm LPF, indicates that the emission is primarily confined to 1250–1300 nm, consistent with the characteristic ^1^O_2_ phosphorescence near 1270 nm (Fig. 4b).

The phosphorescence decay profiles further confirm that this metasurface with sub-micron optical path (100 nm) achieves ^1^O_2_ emission intensity comparable to macroscopic photosensitizer solutions (10 μM Rose Bengal aqueous solution with the optical path as long as 10 mm, Fig. 4c). We chose 10 μM RB as a representative benchmark concentration because increasing the concentration of conventional molecular photosensitizers does not increase singlet oxygen output, owing to concentration-dependent self-quenching/aggregation effects^61,62^. Specifically, the ^1^O_2_ phosphorescence counts from 10 μM Rose Bengal (RB) solution (yellow curve) and from the metasurface (blue curve) exhibit identical peak amplitudes in temporal fitting (Fig. 4c). The measured ^1^O_2_ lifetime for the BIC metasurface is 47 μs, which is shorter than the 63 and 64 μs lifetimes observed for 10 μM and 30 μM RB solutions, respectively. This reduction in lifetime is attributed to the elevated local oxygen concentration near the TiO_2_ surface, where oxygen adsorption significantly enhances the quenching of ^1^O_2_^63–65^. The local ^1^O_2_ concentration at the metasurface interface reaches approximately 1.12 M as calculated (Supplementary Text 5), which is 10^6^ times higher than the ^1^O_2_ concentrations achieved by traditional photosensitizer-based methods (~0.87 μM from RB aqueous solution). This concentration is primarily due to TiO_2_’s strong oxygen adsorption and the photonic enhancement provided by the metasurface.

The wavelength-specific nature of ^1^O_2_ nanogenerator is further confirmed by varying the structural parameter *S*. Metasurfaces with *S* = 1.104 and *S* = 1.106 exhibit significantly higher ^1^O_2_ photon counts relative to the baseline (negative control), while metasurfaces with *S* ≥ 1.108 show no detectable activity (Fig. S3c). This strong dependence on the q-BIC resonance highlights the critical role of photonic mode engineering in driving efficient ^1^O_2_ generation.

### Temporal evolution of singlet oxygen generation

**Fig. 5:**
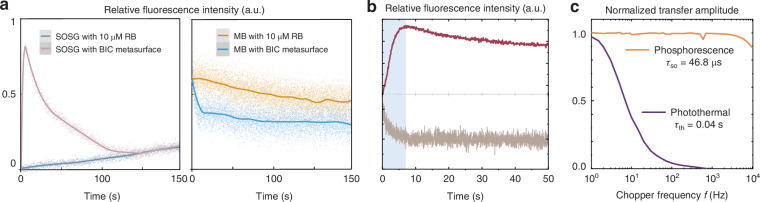
Chemical readouts and frequency-domain discrimination of singlet-oxygen generation. **a** Temporal traces of reporter fluorescence under continuous 532 nm excitation. Left: SOSG probe; right: MB bleaching. In both reporters, the Au–TiO_2_ q-BIC metasurface yields a markedly stronger response than a 10 μM RB reference, confirming efficient ^1^O_2_ production. **b** Illumination on/off test (blue-shaded window indicates the initial light-on period). The photon-count signal rises promptly upon excitation and remains well above the negative control, confirming a light-triggered process. **c** Bode analysis separating photothermal effects from ^1^O_2_ phosphorescence. The normalized transfer amplitude versus chopper frequency shows a flat, high-bandwidth response for the NIR phosphorescence channel (characteristic lifetime *τ*_SO_ ≈ 46.8 μs), while the photothermal signal exhibits a strong low-pass roll-off with a much longer thermal time constant (*τ*_th_ ≈ 0.04 s). The distinct bandwidths demonstrate that the detected ^1^O_2_ emission is not a thermal artifact

To investigate the temporal evolution of ^1^O_2_ generation, we employ singlet oxygen sensor green fluorescent probe (SOSG)^66^, a highly selective fluorescent probe that reacts specifically with ^1^O_2_ to form SOSG-ep while emitting green fluorescence. Under identical conditions with constant incident photon flux, both the BIC metasurface and a 10 μM RB photosensitizer solution are illuminated while monitoring SOSG fluorescence changes to infer ^1^O_2_ generation (Fig. 5a, left). The metasurface exhibits a rapid fluorescence intensity rise within the initial 8 seconds, demonstrating enhanced ^1^O_2_ generation kinetics surpassing conventional photosensitizer solutions. This accelerated dynamics is attributed to the prioritized activation of oxygen molecules adsorbed on TiO_2_ through photoinduced carrier transfer.

Under laser illumination of the RB solution, ^1^O_2_ is produced throughout the entire illuminated region. In contrast, when the laser is focused on the metasurface, the generation of ^1^O_2_ is confined to within a diffusion length of only a few hundred nanometers (~800 nm) above the patterned region. Although the total number of ^1^O_2_ molecules generated in the RB solution and metasurface may become comparable over extended irradiation times (Fig. 5a, left), the distinct volumes in which they exist result in significantly different concentration profiles (Fig. S4). The fluorescence intensity from BIC metasurface then gradually decreased after the peak at 8 s, which can be attributed to the conversion of ^1^O_2_ into superoxide anions (${O}_{2}^{-}$) that subsequently desorb from the TiO_2_ surface^67^. This transformation signifies the completion of the catalytic cycle, where ^1^O_2_ activation leads to oxygen molecule dissociation and regeneration of active sites.

To further confirm the photocatalytic activity of the metasurface, methylene-blue (MB) degradation experiments are conducted (Fig. 5a, right)^12^. Under identical illumination conditions, the metasurface demonstrates faster MB degradation compared to the 10 μM RB solution, validating its superior photocatalytic performance. The enhanced degradation rate correlates with the accelerated ^1^O_2_ generation observed in the SOSG experiments. A direct comparison of the temporal behavior of SOSG fluorescence and MB degradation (Fig. 5b) reveals that the two processes exhibit consistent kinetic profiles under illumination. This temporal alignment reflects the ultrafast generation of ^1^O_2_ by the metasurface immediately after the onset of illumination, providing valuable insights into the dynamics of photonic-enhanced reactive oxygen species (ROS) production in the early stages of light activation.

To exclude the possibility that photothermal effects produce ^1^O_2_, we performed a compact frequency-domain discrimination by modulating the pump light and comparing the normalized transfer amplitudes of the NIR phosphorescence (1270 nm band) with those of the metasurface reflectance measured by a photoelectric detector (Fig. 5c). The phosphorescence exhibits a flat, high-bandwidth response consistent with the microsecond lifetime of ^1^O_2_, whereas the reflectance shows a strong low-pass roll-off with a thermal time constant on the order of 10^−2^ s. The clear bandwidth separation confirms that the detected 1270 nm emission is photochemical rather than photothermal.

### Evaluation of the ability of metasurfaces to induce death in cells

Under short-term laser irradiation, the concentration of ^1^O_2_ generated by photosensitizers dispersed in aqueous environments is generally insufficient to induce cell death, as the threshold concentration required for cell death is approximately in the millimolar range^21^. Achieving higher ^1^O_2_ concentrations requires increasing photosensitizer dosage and extending laser exposure times to several hours. However, excessive photosensitizer levels pose significant toxicity to biological tissues, limiting their biomedical applications^68,69^, and prolonged irradiation significantly increases patient pain^70,71^. Notably, BIC metasurfaces introduced here effectively overcome these limitations, having the ^1^O_2_ concentration rapidly reaching to molar level within 10 s.

To investigate the cytotoxicity of q-BIC metasurfaces through ^1^O_2_ generation, we conducted in vitro experiments using U2OS human osteosarcoma cells cultured directly on metasurface substrates^72^. The substrate comprises a circular SiO_2_ wafer with three identical metasurface modules, each containing parametric arrays defined by geometric parameters (Fig. S6).

**Fig. 6:**
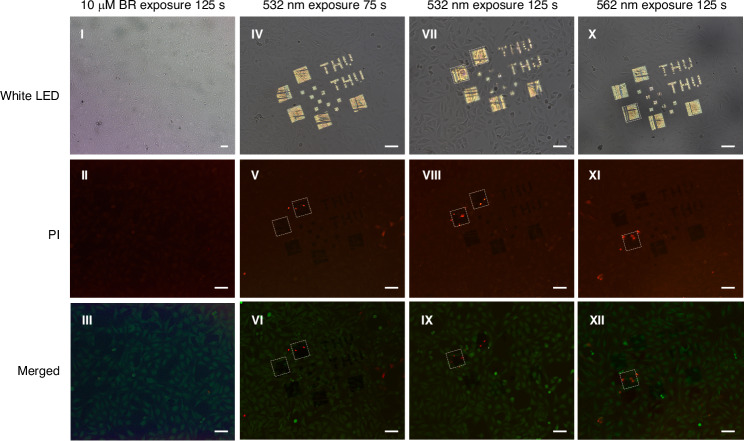
Wavelength-selective, pixel-resolved photocytotoxicity on Au–TiO_2_ q-BIC metasurfaces. Cells were cultured directly on patterned metasurfaces and imaged in three channels: *White LED* (bright field, top row), *PI* (propidium iodide; dead, middle row), and *Merged* (Calcein-AM/PI overlay; live/dead, bottom row). Columns (left to right): **I**–**III** 10 μM RB control, 125 s exposure; **IV**–**VI** 532 nm irradiation for 75 s; **VII**–**IX** 532 nm for 125 s; **X**–**XII** 562 nm for 125 s. Dashed boxes mark representative metasurface pixels used for comparison. Under 532 nm illumination (**IV**–**IX**), PI-positive cells concentrate in resonance-mapped zones corresponding to designs scaled to *S* = 1.104 and *S* = 1.106; surrounding, off-resonant regions retain Calcein fluorescence. When the excitation is shifted to 562 nm (**X**–**XII**), cell death localizes to pixels red-tuned to *S* = 1.112, consistent with spectral matching. Scale bars, 10 μm. Together, these data demonstrate spatially selective, resonance-matched photodynamic cytotoxicity arising from localized singlet-oxygen generation

A 532 nm expanded laser beam (diameter: 2.5 mm, power: 25 mW) uniformly irradiates each metasurface module. Post-exposure samples are incubated for 6 h to allow death progression, with cell death assessed by Calcein-AM/PI dual staining. Apoptotic cells are mainly localized around metasurface-patterned regions *S* = 1.104 and *S* = 1.106 (Fig. 6, Panels V and VIII). Dose-response analysis reveals time dependence: increasing exposure duration from 75 to 125 s enhances ^1^O_2_ generation, correlating with elevated cell mortality.

This spatial selectivity aligns with spectral response mapping (Fig. 3h), where regions of *S* = 1.104 and *S* = 1.106 exhibit stronger 532 nm resonance than regions of *S* = 1.108–1.112, demonstrating the localized cytotoxicity. To validate wavelength specificity, the 532 nm laser is replaced with a 562 nm light source (25 mW, 125 s exposure), which induces significant cell death near region of *S* = 1.112 (Fig. 6a, Panel XI), corresponding to the 562 nm reflectance peak. In contrast, using 10 μM RB in Hank’s balanced salt solution (HBSS) buffer under equivalent photodynamic conditions (25 mW, 125 s exposure, Fig. 6a, Panels I–III) shows no patterned cell death in laser-irradiated regions.

The above results demonstrate that q-BIC metasurfaces enable precision-targeted photodynamic therapy, with spatial resolution governed by metasurface patterns and spectral selectivity defined by engineered optical resonances. This on-demand ^1^O_2_ generation technology achieves unprecedented spatiotemporal control, representing a significant advancement over traditional photosensitizer-based cell-targeting approaches.

## Discussion

We have established a metasurface strategy that unites q-BIC resonance control with critical coupling to maximize interfacial absorption in a minimal-metal Au-TiO_2_ architecture. By balancing radiative leakage with intrinsic loss within a subwavelength optical path, the q-BIC metasurface concentrates optical energy at Au/TiO_2_ heterojunctions while maintaining ultralow noble-metal loading. The small metal volume concentrates the same absorbed power at high density, thereby elevating the electron temperature and strengthening the hot-carrier population; simultaneously, the reduced density of metallic recombination centers suppresses carrier recombination and extends carrier persistence at the interface. This design yields strong absorption at 532 nm and promotes hot-carrier generation and longevity, enabling local molar-level ^1^O_2_ within seconds and an approximately six-order-of-magnitude increase in local ^1^O_2_ concentration compared with conventional approaches, providing a material-efficient route to activate interfacial redox chemistry.

Under continuous-wave excitation, near-interface photochemistry rapidly yields pixel-confined, high-density ^1^O_2_, enabling on-chip spatially programmable cytotoxicity. The ^1^O_2_ signal is validated by time-resolved detection of the 1270 nm phosphorescence with spectral filtering, and complementary chemical readouts (SOSG activation and MB bleaching) exhibit temporally coherent kinetics, confirming a common reactive-oxygen flux. The metasurface thereby enables wavelength- and pixel-level control of reactivity without molecular sensitizers, yielding rapid, spatially resolved cytotoxicity in cell assays.

Beyond the specific Au–TiO_2_ implementation, the principles demonstrated here—q-BIC mode engineering, impedance matching via critical-coupling, and minimal-metal hot-carrier activation—can be applied to other metal/semiconductor pairs and spectral bands. In stark contrast to conventional dye sensitizers and many nanoparticle photocatalysts that often require higher dosages and prolonged illumination and tend to be inadvertently activated by ambient light due to broadband absorption, our metasurface enables rapid build-up of highly localized ^1^O_2_ with wavelength-addressable, patterned reactivity. We anticipate immediate opportunities in rapid photodynamic therapy, selective oxidation, and on-chip or flow microreactors, where sensitizer-free operation, reduced cross-talk, and low noble-metal usage are desirable. Future work will focus on extending operation to NIR excitation, optimizing interface chemistry and stability in complex media, and integrating multiplexed resonators for spatial/spectral programmability of photon-to-chemical conversion at solid–liquid interfaces.

## Materials and methods

### Device fabrication

Au–TiO_2_ q-BIC metasurfaces are fabricated on BF33 float-glass substrates. Substrates are sonicated in acetone and ethanol for 10 min at 200 W, rinsed with deionized water, dried with N_2_, and baked at 120 °C for 30 min. A 950 PMMA A4 resist is spin-coated at 3000 rpm for 45 s and baked at 180 °C for 90 s. Patterns are defined by electron-beam lithography (NB5, 80 kV, 0.8 nA, dose 8 C/m^2^) under high vacuum (<2.7e-8 Torr), developed in MIBK:IPA = 1:3 for 60 s, rinsed in IPA for 60 s, and dried with N_2_. Films are deposited by e-beam evaporation in a PVD75 (<1e-7 Torr, 1 Å/s): 100 nm TiO_2_ followed by 7 nm Au. Lift-off in acetone for 48 h yields Au-capped TiO_2_ elliptical nanopillars.

### Numerical modeling

A single unit cell is modeled in COMSOL Multiphysics using Floquet periodic boundary conditions in the in-plane directions and perfectly matched layers along the propagation axis. The top and bottom faces use scattering boundaries. The superstrate is deuterated water and the substrate is SiO_2_. The geometry consists of a 100 nm TiO_2_ pillar capped by a 7 nm Au film.

### Time-correlated single-photon counting of ^1^O_2_

Singlet-oxygen phosphorescence at 1270 nm is measured with a single-photon detector (Aurea Technology, SPD_NIR, 900-1700 nm) time-tagged by a time-correlated single-photon counting module (PicoQuant TimeHarp 260 NANO). Samples are excited with a pulsed 532 nm laser (pulse width of 35 μs, repetition rate of 2 kHz). Emission is collected through a Nikon TU PLAN ELWD 50×/0.60 long-working-distance objective in the same microscope path; LPFs at 1250 and 1300 nm, together with a 532 nm notch filter, block the 532 nm excitation. Removal of the 1300 nm LPF verifies that the detected signal lies within 1250–1300 nm.

### Frequency-domain discrimination

The pump light is mechanically modulated with an optical chopper (Thorlabs MC2000B; modulation frequency up to 10 kHz). The amplitude transfer response of the 1270 nm phosphorescence channel is compared with a simultaneously recorded reflectance channel detected by a photodiode amplifier (Thorlabs PDA36A2; 350–1100 nm). The phosphorescence channel shows a flat, high-bandwidth response consistent with microsecond-lived emission, whereas the reflectance exhibits a pronounced low-pass roll-off with a thermal time constant of about 0.04 s, confirming a photochemical origin of the NIR signal.

### Photon-flux normalization

To avoid ambiguity between incident and absorbed photon budgets, we explicitly distinguish the incident photon flux *Φ*_inc_ and absorbed photon flux *Φ*_abs_ in data analysis. The incident optical power *P*_inc_ is measured at the sample plane using a calibrated power meter. The corresponding incident photon flux at wavelength *λ* is calculated as

2 $${\Phi }_{{\rm{i}}{\rm{n}}{\rm{c}}}(\lambda )=\frac{{P}_{{\mathrm{inc}}}}{hc/\lambda }=\frac{{P}_{{\mathrm{inc}}}\lambda }{hc}$$

where *h* is Planck’s constant and *c* is the speed of light. When photon-flux *density* is needed, we have

3 $${\phi }_{{\mathrm{inc}}}(\lambda )=\frac{{\Phi }_{{\mathrm{inc}}}({\rm{\lambda }})}{S}$$

where *S* is the illuminated area at the sample.

The absorbed photon flux is obtained from the measured absorptance *A*(*λ*) as

4 $${\Phi }_{{\mathrm{abs}}}({\rm{\lambda }})={\Phi }_{{\mathrm{inc}}}({\rm{\lambda }})\times A({\rm{\lambda }})$$

and the absorbed optical power is *P*_abs_(λ) = *P*_inc_(λ) × *A*(λ). The absorptance is determined from reflection and transmission measurements via

5 $$A(\lambda )=1-R({\rm{\lambda }})-T({\rm{\lambda }})$$

where *R*(λ) and *T*(λ) are the total reflectance and transmittance (measured, e.g., with a spectrophotometer equipped with an integrating sphere). Unless otherwise stated, kinetic traces (e.g., SOSG activation, MB bleaching, and 1270 nm phosphorescence counting) are acquired and plotted under fixed incident power *P*_inc_ (i.e., constant *Φ*_inc_). In contrast, any quantity intended to represent an intrinsic per-photon efficiency (e.g., apparent quantum-yield-type metrics) is normalized to the absorbed photon flux Φ_abs_ using the corresponding *A*(*λ*).

### Chemical reporters

SOSG stock is prepared by dissolving 100 μg reagent in 33 μl methanol (about 5 mM), then diluting to 10 μM with deionized water before applying to the metasurface under a water layer. A non-patterned area with 10 μM RB serves as a blank, and four samplings are averaged. An MB working solution is prepared at 10 μM. MB bleaching and SOSG fluorescence are recorded under continuous 532 nm excitation in the same microscope to track ^1^O_2_ dynamics.

### Cell culture and phototoxicity

U2OS human osteosarcoma cells are cultured directly on the metasurface at 37 °C in a CO_2_ incubator. Experiments start after the cell adhesion. Live/dead staining reagents (Calcein-AM and propidium iodide, PI) are added, and the cells are incubated for 6 h. The entire patterned area is then irradiated by a 532 nm (or 562 nm) laser for the indicated duration, after which the cells are returned to the incubator for an additional 30 min. Cell death is assessed on a fluorescence microscope (Olympus BX53M) using an Olympus U-HGLGPS 130 W fiber-coupled fluorescence light source for excitation.

## Supplementary information

Supplementary Material for Quasi-BICmetasurfacesenablerapid, localizedsinglet-oxygengeneration

## Data Availability

All data needed to evaluate the conclusions in the paper are present in the paper and supplementary material. Additional data related to this paper may be requested from the authors.

## References

[1] DeRosa, M. C. & Crutchley, R. J. Photosensitized singlet oxygen and its applications. *Coord. Chem. Rev.* **233–234**, 351–371 (2002).

[2] Schweitzer, C. & Schmidt, R. Physical mechanisms of generation and deactivation of singlet oxygen. *Chem. Rev.* **103**, 1685–1758 (2003). 12744692 10.1021/cr010371d

[3] Ogilby, P. R. Singlet oxygen: there is indeed something new under the sun. *Chem. Soc. Rev.* **39**, 3181–3209 (2010). 20571680 10.1039/b926014p

[4] Blázquez-Castro, A. Direct ^1^O_2_ optical excitation: a tool for redox biology. *Redox Biol.* **13**, 39–59 (2017). 28570948 10.1016/j.redox.2017.05.011 PMC5451181

[5] Dougherty, T. J. et al. Photodynamic therapy. *J. Natl. Cancer Inst.* **90**, 889–905 (1998). 9637138 10.1093/jnci/90.12.889 PMC4592754

[6] Agostinis, P. et al. Photodynamic therapy of cancer: an update. *CA Cancer J. Clin.* **61**, 250–281 (2011). 21617154 10.3322/caac.20114 PMC3209659

[7] Zhao, J. & Yang, X. D. Photocatalytic oxidation for indoor air purification: a literature review. *Build. Environ.* **38**, 645–654 (2003).

[8] Mamaghani, A. H., Haghighat, F. & Lee, C. S. Photocatalytic oxidation technology for indoor environment air purification: the state-of-the-art. *Appl. Catal. B Environ.* **203**, 247–269 (2017).

[9] Wang, Y. et al. Singlet oxygen: Properties, generation, detection, and environmental applications. *J. Hazard. Mater.* **461**, 132538 (2024). 37734310 10.1016/j.jhazmat.2023.132538

[10] Kou, J. Y., Dou, D. & Yang, L. M. Porphyrin photosensitizers in photodynamic therapy and its applications. *Oncotarget* **8**, 81591–81603 (2017). 29113417 10.18632/oncotarget.20189 PMC5655312

[11] Ferreira, B. C. L. B. et al. Effect of concentration on singlet oxygen generation from xanthene-based photosensitizers. *J. Photochem. Photobiol. A Chem.* **461**, 116167 (2025).

[12] Chen, P. et al. Synchronous photosensitized degradation of methyl orange and methylene blue in water by visible-light irradiation. *J. Mol. Liq.* **334**, 116159 (2021).

[13] Zhao, J. et al. Triplet photosensitizers: from molecular design to applications. *Chem. Soc. Rev.* **42**, 5323–5351 (2013). 23450221 10.1039/c3cs35531d

[14] Kamkaew, A. et al. Bodipy dyes in photodynamic therapy. *Chem. Soc. Rev.* **42**, 77–88 (2013). 23014776 10.1039/c2cs35216h PMC3514588

[15] Xing, C. F. et al. Conjugated polymer/porphyrin complexes for efficient energy transfer and improving light-activated antibacterial activity. *J. Am. Chem. Soc.* **131**, 13117–13124 (2009). 19702260 10.1021/ja904492x

[16] Abrahamse, H. & Hamblin, M. R. New photosensitizers for photodynamic therapy. *Biochem. J.* **473**, 347–364 (2016). 26862179 10.1042/BJ20150942 PMC4811612

[17] Younis, M. R. et al. Inorganic nanomaterials with intrinsic singlet oxygen generation for photodynamic therapy. *Adv. Sci.* **8**, 2102587 (2021). 10.1002/advs.202102587 PMC8564446 34561971

[18] Pasparakis, G. Light-induced generation of singlet oxygen by naked gold nanoparticles and its implications to cancer cell phototherapy. *Small* **9**, 4130–4134 (2013). 23813944 10.1002/smll.201301365

[19] Cheng, Y. et al. Simulated sunlight-mediated photodynamic therapy for melanoma skin cancer by titanium-dioxide-nanoparticle–gold-nanocluster–graphene heterogeneous nanocomposites. *Small* **13**, 1603935 (2017). 10.1002/smll.201603935 28371113

[20] Chen, Y. et al. Kissing-loop nano-kirigami structures with asymmetric transmission and anomalous reflection. *Light Adv. Manuf.* **5**, 498–508 (2025).

[21] Zhu, T. C. et al. Comparison of singlet oxygen threshold dose for PDT. In Proc. *SPIE 8931, Optical Methods for Tumor Treatment and Detection: Mechanisms and Techniques in Photodynamic Therapy XXIII* (SPIE, 2014). 10.1117/12.2039719 PMC4437725 25999651

[22] Zhao, R. F. et al. Photothermal effect enhanced cascade-targeting strategy for improved pancreatic cancer therapy by gold nanoshell@mesoporous silica nanorod. *ACS Nano* **11**, 8103–8113 (2017). 28738680 10.1021/acsnano.7b02918

[23] Gellé, A. et al. Enhancing singlet oxygen photocatalysis with plasmonic nanoparticles. *ACS Appl. Mater. Interfaces* **13**, 35606–35616 (2021). 34309350 10.1021/acsami.1c05892

[24] Park, J. et al. Physically-synthesized gold nanoparticles containing multiple nanopores for enhanced photothermal conversion and photoacoustic imaging. *Nanoscale* **8**, 15514–15520 (2016). 27527067 10.1039/c6nr05376a

[25] Aioub, M., Panikkanvalappil, S. R. & El-Sayed, M. A. Platinum-coated gold nanorods: efficient reactive oxygen scavengers that prevent oxidative damage toward healthy, untreated cells during plasmonic photothermal therapy. *ACS Nano* **11**, 579–586 (2017). 28029783 10.1021/acsnano.6b06651

[26] Nosaka, Y. et al. Singlet oxygen formation in photocatalytic TiO_2_ aqueous suspension. *Phys. Chem. Chem. Phys.* **6**, 2917–2918 (2004).

[27] Li, Y. et al. Mechanism of photogenerated reactive oxygen species and correlation with the antibacterial properties of engineered metal-oxide nanoparticles. *ACS Nano* **6**, 5164–5173 (2012). 22587225 10.1021/nn300934k

[28] Daimon, T. & Nosaka, Y. Formation and behavior of singlet molecular oxygen in tio2 photocatalysis studied by detection of near-infrared phosphorescence. *J. Phys. Chem. C.* **111**, 4420–4424 (2007).

[29] Li, X. H., Zhu, J. M. & Wei, B. Q. Hybrid nanostructures of metal/two-dimensional nanomaterials for plasmon-enhanced applications. *Chem. Soc. Rev.* **45**, 3145–3187 (2016). 27048993 10.1039/c6cs00195e

[30] Yang, D. et al. Au nanoclusters sensitized black TiO_2−*x*_ nanotubes for enhanced photodynamic therapy driven by near-infrared light. *Small* **13**, 1703007 (2017). 10.1002/smll.201703007 29094517

[31] He, W. W. et al. Photogenerated charge carriers and reactive oxygen species in ZnO/Au hybrid nanostructures with enhanced photocatalytic and antibacterial activity. *J. Am. Chem. Soc.* **136**, 750–757 (2014). 24354568 10.1021/ja410800y

[32] Vankayala, R., Sagadevan, A., Vijayaraghavan, P., Kuo, C.-L. & Hwang, K. C. Metal nanoparticles sensitize the formation of singlet oxygen. *Angew. Chem. Int. Ed.* **50**, 10640 (2011). 10.1002/anie.201105236 21932210

[33] Gao, F. et al. A singlet oxygen generating agent by chirality-dependent plasmonic shell-satellite nanoassembly. *Adv. Mater.* **29**, 1606864 (2017). 10.1002/adma.201606864 28230915

[34] Hsu, C. W. et al. Bound states in the continuum. *Nat. Rev. Mater.* **1**, 16048 (2016).

[35] Kang, M. et al. Applications of bound states in the continuum in photonics. *Nat. Rev. Phys.* **5**, 659–678 (2023).

[36] Koshelev, K. et al. Asymmetric metasurfaces with high-Q resonances governed by bound states in the continuum. *Phys. Rev. Lett.* **121**, 193903 (2018). 30468599 10.1103/PhysRevLett.121.193903

[37] Liu, T., Guo, C., Li, W. & Fan, S. Thermal photonics with broken symmetries. *ELight* **2**, 25 (2022).

[38] Pavlov, D. V. et al. Nonlinear light conversion and infrared photodetection with laser-printed plasmonic metasurfaces supporting bound states in the continuum. *Light Sci. Appl.* **15**, 23 (2026). 41478853 10.1038/s41377-025-02040-4 PMC12757600

[39] Rao, L. X. et al. Meron spin textures in momentum space spawning from bound states in the continuum. *Phys. Rev. Lett.* **135**, 026203 (2025). 40743166 10.1103/3g3j-mnh9

[40] Maggiolini, E. et al. Strongly enhanced light–matter coupling of monolayer WS_2_ from a bound state in the continuum. *Nat. Mater.* **22**, 964–969 (2023). 37217703 10.1038/s41563-023-01562-9

[41] Tseng, M. L. et al. Dielectric metasurfaces enabling advanced optical biosensors. *ACS Photonics* **8**, 47–60 (2021).

[42] Dong, Z. G. et al. Nanoscale mapping of optically inaccessible bound-states-in-the-continuum. *Light Sci. Appl.* **11**, 20 (2022). 35058424 10.1038/s41377-021-00707-2 PMC8776833

[43] Tittl, A. et al. Imaging-based molecular barcoding with pixelated dielectric metasurfaces. *Science* **360**, 1105–1109 (2018). 29880685 10.1126/science.aas9768

[44] Aigner, A. et al. Continuous spectral and coupling-strength encoding with dual-gradient metasurfaces. *Nat. Nanotechnol.* **19**, 1804–1812 (2024). 39187580 10.1038/s41565-024-01767-2 PMC11638065

[45] Hu, H. Y. et al. Catalytic metasurfaces empowered by bound states in the continuum. *ACS Nano* **16**, 13057–13068 (2022). 35953078 10.1021/acsnano.2c05680 PMC9413421

[46] Yang, S. & Ndukaife, J. C. Optofluidic transport and assembly of nanoparticles using an all-dielectric Quasi-BIC metasurface. *Light Sci. Appl.* **12**, 188 (2023). 37507389 10.1038/s41377-023-01212-4 PMC10382587

[47] Crotti, G. et al. Ultrafast switching of a metasurface quasi-bound state in the continuum via transient optical symmetry breaking. *Light Sci. Appl.* **14**, 240 (2025). 40623993 10.1038/s41377-025-01885-z PMC12234965

[48] Jin, R. et al. Toroidal dipole BIC-driven highly robust perfect absorption with a graphene-loaded metasurface. *Nano Lett.* **23**, 9105–9113 (2023). 37694889 10.1021/acs.nanolett.3c02958

[49] Jiang, X. Y. et al. Tunable ultra-high-efficiency light absorption of monolayer graphene using critical coupling with guided resonance. *Opt. Express* **25**, 27028–27036 (2017). 29092184 10.1364/OE.25.027028

[50] Ostovar, B. et al. The role of the plasmon in interfacial charge transfer. *Sci. Adv.* **10**, eadp3353 (2024). 38968358 10.1126/sciadv.adp3353 PMC11225779

[51] Yuan, L. et al. Metal-semiconductor heterostructures for photoredox catalysis: where are we now and where do we go?. *Adv. Funct. Mater.* **31**, 2101103 (2021).

[52] Taghinejad, M. et al. Determining hot-carrier transport dynamics from terahertz emission. *Science* **382**, 299–305 (2023). 37856614 10.1126/science.adj5612

[53] Xiao, Q. et al. Hot-carrier organic synthesis via the near-perfect absorption of light. *ACS Catal.* **8**, 10331–10339 (2018).

[54] Sun, S. et al. Nanoparticle loading effects on the broadband absorption for plasmonic-metal@semiconductor-microsphere photocatalyst. *Catal. Today* **278**, 312–318 (2016).

[55] Shaviv, E. et al. Absorption properties of metal-semiconductor hybrid nanoparticles. *ACS Nano* **5**, 4712–4719 (2011). 21648441 10.1021/nn200645h

[56] Tang, J. Q. et al. Calculation extinction cross sections and molar attenuation coefficient of small gold nanoparticles and experimental observation of their UV-vis spectral properties. *Spectrochim. Acta Part A Mol. Biomol. Spectrosc.* **191**, 513–520 (2018). 10.1016/j.saa.2017.10.047 29091910

[57] Dolinnyi, A. I. Extinction coefficients of gold nanoparticles and their dimers. dependence of optical factor on particle size. *Colloid J.* **79**, 611–620 (2017).

[58] Massicotte, M. et al. Photo-thermionic effect in vertical graphene heterostructures. *Nat. Commun.* **7**, 12174 (2016). 27412308 10.1038/ncomms12174 PMC4947168

[59] Saito, H. & Nosaka, Y. Mechanism of singlet oxygen generation in visible-light-induced photocatalysis of gold-nanoparticle-deposited titanium dioxide. *J. Phys. Chem. C* **118**, 15656–15663 (2014).

[60] Morozov, P. et al. Singlet oxygen phosphorescence imaging by superconducting single-photon detector and time-correlated single-photon counting. *Opt. Lett.* **46**, 1217–1220 (2021). 33720151 10.1364/OL.415229

[61] Peterson, J. C. et al. Detection of singlet oxygen luminescence for experimental corneal rose bengal photodynamic antimicrobial therapy. *Biomed. Opt. Express* **12**, 272–287 (2020). 33520385 10.1364/BOE.405601 PMC7818961

[62] Mokrzyński, K. & Szewczyk, G. The (un) known issue with using rose bengal as a standard of singlet oxygen photoproduction. *Photochem. Photobiol.* **101**, 546–549 (2025). 39377455 10.1111/php.14030

[63] Nonell, S. & Flors, C. *Singlet Oxygen: Applications in Biosciences and Nanosciences* (Royal Society of Chemistry, 2016).

[64] Hatz, S., Poulsen, L. & Ogilby, P. R. Time-resolved singlet oxygen phosphorescence measurements from photosensitized experiments in single cells: effects of oxygen diffusion and oxygen concentration. *Photochem. Photobiol.* **84**, 1284–1290 (2008). 18435700 10.1111/j.1751-1097.2008.00359.x

[65] Chen, L. C. et al. Effect of oxygen concentration on singlet oxygen luminescence detection. *J. Lumin.* **152**, 98–102 (2014).

[66] Gollmer, A. et al. Singlet oxygen sensor green®: photochemical behavior in solution and in a mammalian cell. *Photochem. Photobiol.* **87**, 671–679 (2011). 21272007 10.1111/j.1751-1097.2011.00900.x

[67] Badmus, K. O. et al. Synthesis of oxygen deficient TiO_2_ for improved photocatalytic efficiency in solar radiation. *Catalysts* **11**, 904 (2021).

[68] Ziegler, V. et al. Photosensitizer adhered to cell culture microplates induces phototoxicity in carcinoma cells. *BioMed. Res. Int.* **2013**, 549498 (2013). 23509741 10.1155/2013/549498 PMC3591219

[69] Kulikov, O. A. et al. Phototoxicity in vitro and safety in vivo of the emulsion photosensitizer based on furanocoumarins of heracleum sosnowskyi. *Eur. J. Pharm. Biopharm.* **198**, 114257 (2024). 38479564 10.1016/j.ejpb.2024.114257

[70] Tanew, A. et al. Impact of light dose and fluence rate on the efficacy and tolerability of topical 5-ala photodynamic therapy for actinic keratoses: a randomized, controlled, observer-blinded intrapatient comparison study. *J. Eur. Acad. Dermatol. Venereol.* **39**, 1460–1467 (2025). 39737551 10.1111/jdv.20527 PMC12291031

[71] Ang, J. M. et al. Photodynamic therapy and pain: a systematic review. *Photodiagnosis Photodyn. Ther.* **19**, 308–344 (2017). 28716738 10.1016/j.pdpdt.2017.07.002

[72] Niforou, K. M. et al. The proteome profile of the human osteosarcoma U2OS cell line. *Cancer Genom. Proteom.* **5**, 63–78 (2008). 18359981

