# Supplementary material for: Quasi-BIC metasurfaces enable rapid, localized singlet-oxygen generation
Source: Light Sci Appl. 2026 Apr 3;15:188. doi: 10.1038/s41377-026-02267-9 (PMC13049180; doi:10.1038/s41377-026-02267-9)
Supplement: Supplementary file 1 — Supplementary Material for Quasi-BICmetasurfacesenablerapid, localizedsinglet-oxygengeneration [file 41377_2026_2267_MOESM1_ESM.docx]

Supplementary Material for:

Quasi-BIC metasurfaces enable rapid, localized singlet-oxygen generation

Ruilin Long,^1,2,3^ Laifu Lin,^1,2,3^ Xinwen Qi,^4^ Qiang Liu,^1,2,3*^ Xing Fu^1,2,3*^

^1^Department of Precision Instrument, Tsinghua University, Beijing 100084, China

^2^State Key Laboratory of Precision Space-Time Information Sensing Technology, Beijing 100084, China

^3^Key Laboratory of Photonic Control Technology (Tsinghua University), Ministry of Education, Beijing 100084, China

^4^Department of Chemistry, MOE Key Laboratory of Bioorganic Phosphorus Chemistry and Chemical Biology, Tsinghua University, 100084 Beijing, China.

∗To whom correspondence should be addressed;

fuxing@tsinghua.edu.cn; qiangliu@tsinghua.edu.cn

Figure S1. Geometric structure for optimizing the resonance wavelength of the BIC surface to 532 nm

Figure S2. Normalized thermal field distribution of a single-period structure of a metal-semiconductor hetero-surface

Figure S3. Optical setup for singlet oxygen phosphorescence detection

Figure S4. Experimental setup and geometric parameters for singlet oxygen generation measurements

Figure S5. *In vitro* culture of U2OS human osteosarcoma cells on an Au-TiO₂ optical metasurface.

Figure S6. Post-fabrication characterization of geometric parameter distribution and substrate morphology

Figure S7: Cycling stability and the effect of cell-handling on the ^1^O_2_ phosphorescence signal.

Supplementary Table 1 Effect of particle size on maximum wavelength of absorbance and molar extinction coefficient of gold nanoparticles

Supplementary Text 1 Determination of the critical-coupling angle via TCMT

Supplementary Text 2 Mechanism of Enhanced Photon Absorption in Quasi-BIC Metasurfaces

Supplementary Text 3 Fabrication method of Au-TiO_2_ Metasurfaces

Supplementary Text 4 Calculation of the Heterojunction Current in the Au-TiO_2_ System

Supplementary Text 5 Calculation of Singlet Oxygen Concentration and Quantum Yield

Supplementary Text 6 Protocol for the Preparation of Singlet Oxygen Sensor Green (SOSG) Solution


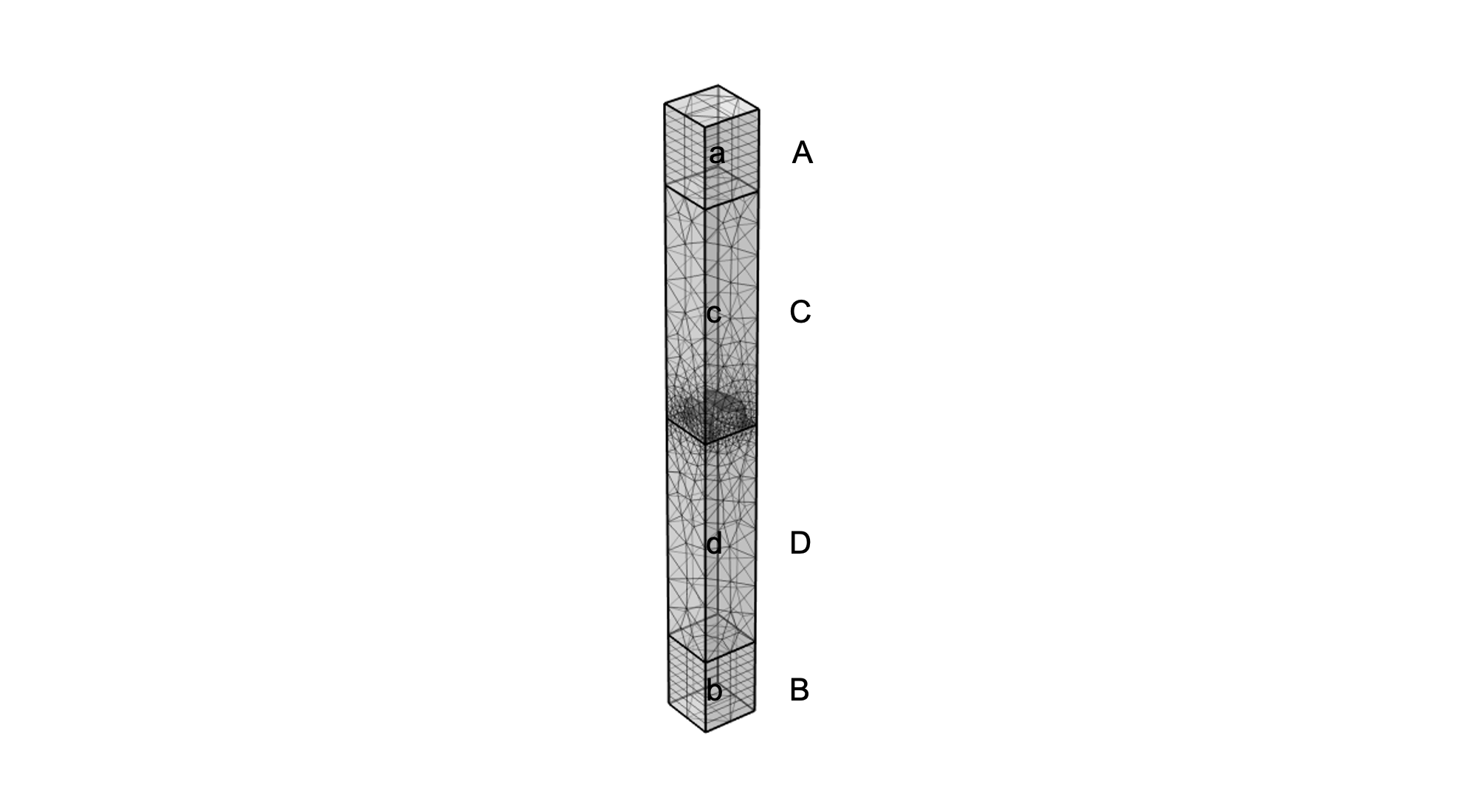


Figure S1: **Geometric structure defined for optimizing the resonance wavelength of the BIC surface to 532 nm.** The bound state in the continuum (BIC) unit cell is modeled numerically following the specified geometric configuration. A single unit cell is simulated and extended to a full-space representation using Floquet periodic boundary conditions, effectively reducing computational complexity while maintaining physical accuracy. As illustrated, rectangular domains **A** and **B** are set as perfectly matched layers (PMLs) to absorb outgoing waves, while top and bottom surfaces **a** and **b** are assigned scattering boundary conditions to minimize undesired reflections. Material properties are applied using COMSOL’s native material library: the upper medium (Region **C**) is modeled as an aqueous environment, while the lower substrate (Region **D**) is defined as silicon dioxide (SiO₂). A 7-nm gold (Au) film is deposited atop a 100-nm titanium dioxide (TiO₂) dielectric pillar to form a metal-dielectric hybrid nanostructure.


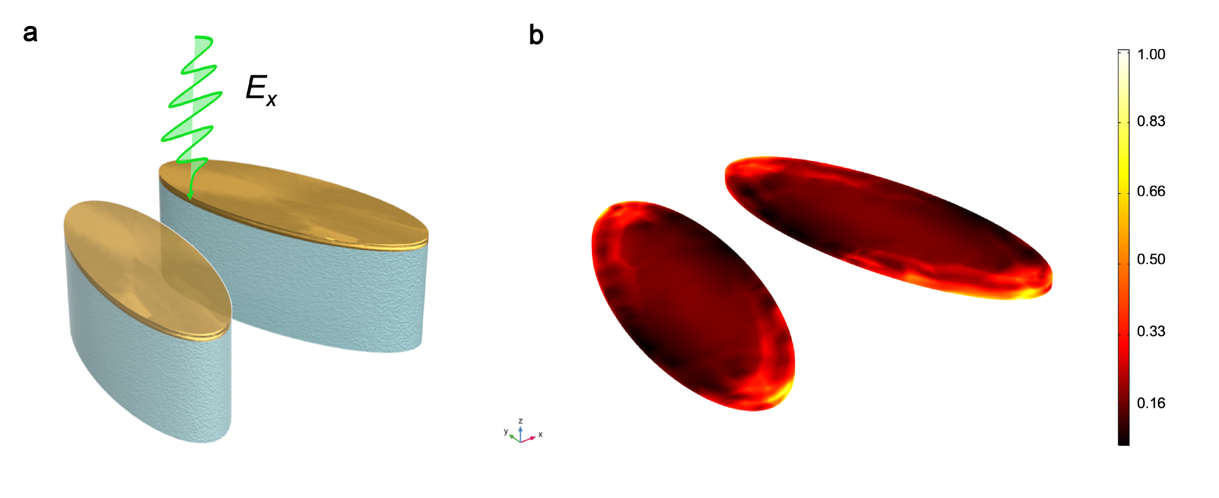


Figure S2: **Normalized thermal field distribution of a single-period structure of a metal-semiconductor hetero-surface.** The dipole mode of BIC typically results in a stronger electric field distribution and localized heating at the two ends of the elliptical structure. We propose that the distribution of the squared electric field mode is consistent with the metal heating distribution^1,2^. According to the simulation, the volume of the BIC mode accounts for 13.49% of the total volume of the metal film. **(a)** Schematic representation of the single-period structure, illustrating the electric field $E_{x}$. **(b)** Simulated normalized thermal field distribution of the structure, showing localized heating at the two ends of the ellipse.


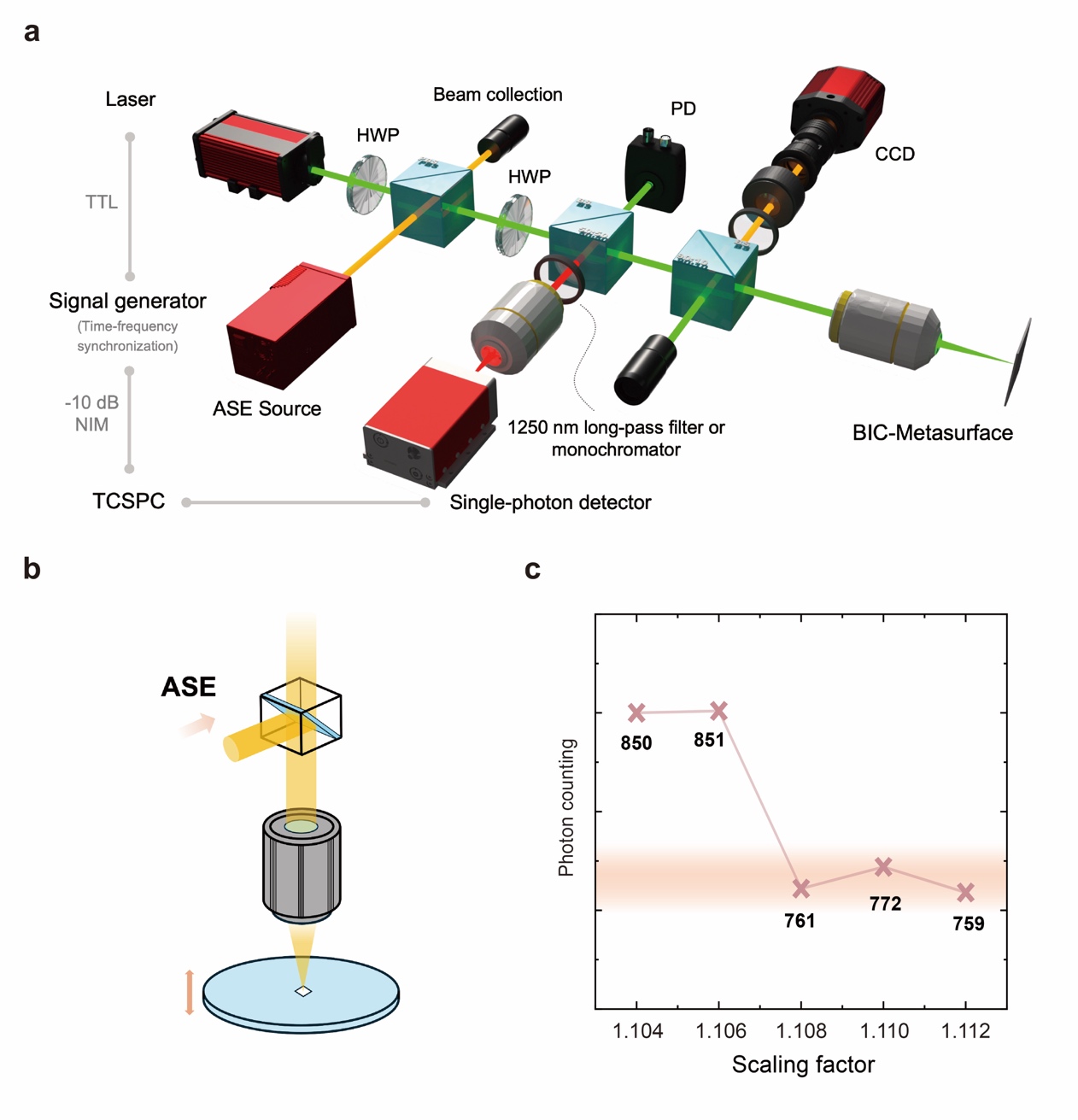


Figure S3: **Optical setup for singlet oxygen phosphorescence detection.** **(a)** Schematic of the phosphorescence detection optical path, which employs a beam splitter configuration to enable both excitation and collection with the same set of equipment. A supercontinuum laser source (ASE, Wuhan Yangtze Soton Laser Co., Ltd., 400-1320 nm spectral range) is used for illumination. The singlet oxygen phosphorescence emitted from the metasurface is collected by the same objective lens and directed to the single-photon detector (Aurea Technology, SPD_NIR, 900-1700 nm) via a time-correlated single-photon counter (TCSPC, PicoQuant TimeHarp 260 NANO) connected through BNC cables. PD: photodetector (Thorlabs, PDA10A2). HWP: 532 nm half-wave plate. CCD: charge-coupled device camera. **(b)** Schematic illustration of the focused supercontinuum laser excitation on the metasurface within aqueous immersion conditions. By vertically adjusting the sample stage, the beam waist is aligned with the metasurface plane to maximize excitation efficiency within the BIC resonance region. **(c)** Photon counting results for metasurfaces with varying scaling factors ($S$). The red error band represents the blank control range, indicating that metasurfaces of $S$= 1.104 and $S$= 1.106 produce ^1^O_2_ signals significantly above the control level, while those of $S$= 1.108-1.112 fall within the error range.

**
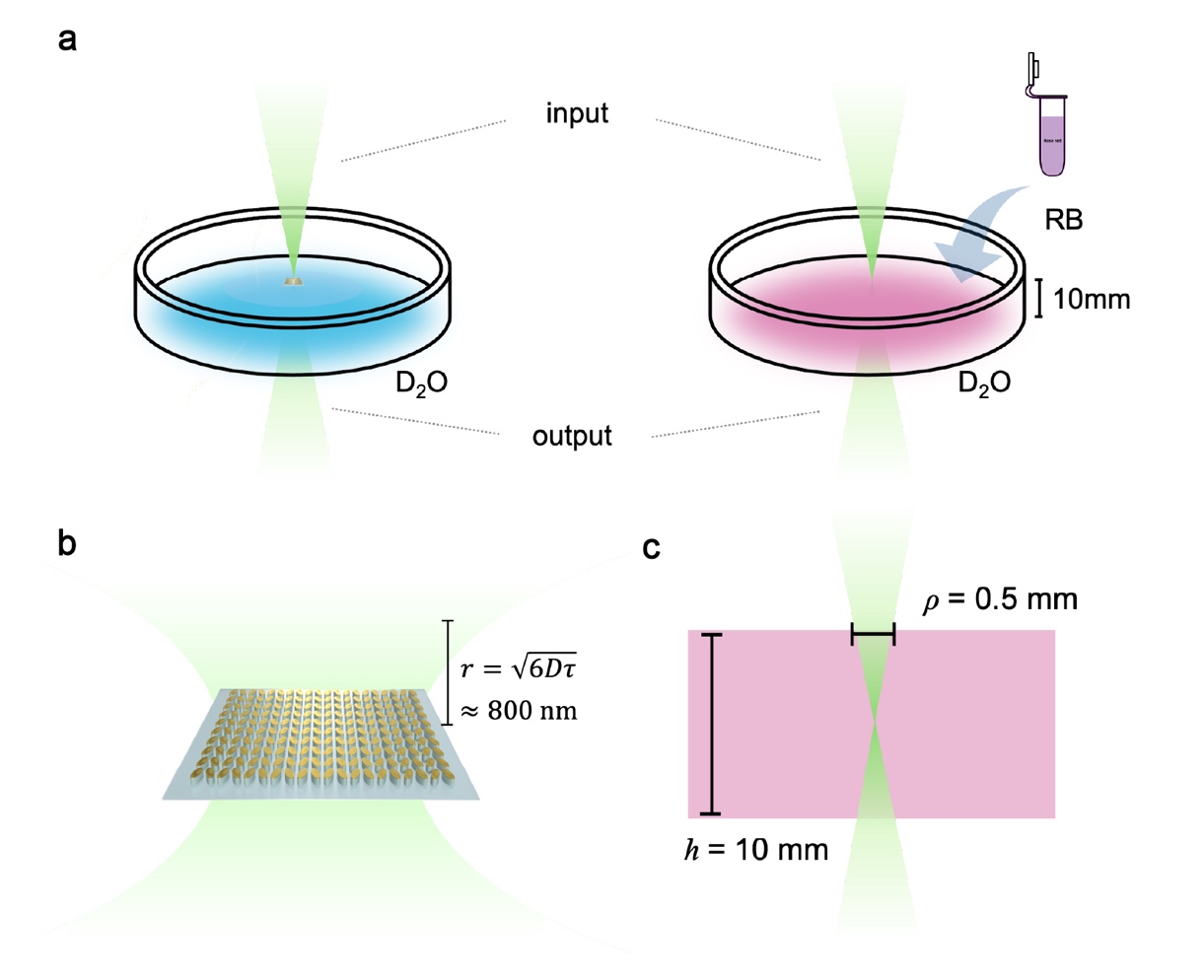
**

Figure S4: **Experimental setup and geometric parameters for singlet oxygen generation measurements. (a)** Schematic of the experimental configuration for singlet oxygen (¹O₂) generation. The left panel illustrates the BIC metasurface immersed in deuterated water (D₂O), where the localized generation of ¹O₂ occurs within a small reaction volume near the metasurface. The right panel shows the reference system using a 10 mm path length of Rose Bengal (RB) solution in D₂O for macroscopic ¹O₂ generation. **(b)** Magnified view of the BIC metasurface, highlighting the localized diffusion radius ($r\approx800$nm) of singlet oxygen, calculated using the diffusion coefficient ($D$) and lifetime ($\tau$) of ¹O₂ in D₂O. **(c)** Geometric comparison of the effective reaction volumes. The metasurface system confines ¹O₂ generation to a small reaction volume ($\rho=0.5$ mm), while the RB solution generates ¹O₂ uniformly across a 10 mm optical path length ($h=10$mm). This setup enables direct comparison of localized versus macroscopic ¹O₂ generation efficiencies. The 10 mm optical path length is used as a standard macroscopic reference, while the comparison metric is the steady-state ¹O₂ concentration in the effective reaction volume (not the total number of generated molecules), making the benchmark insensitive to the absolute liquid-layer thickness under matched focusing conditions.


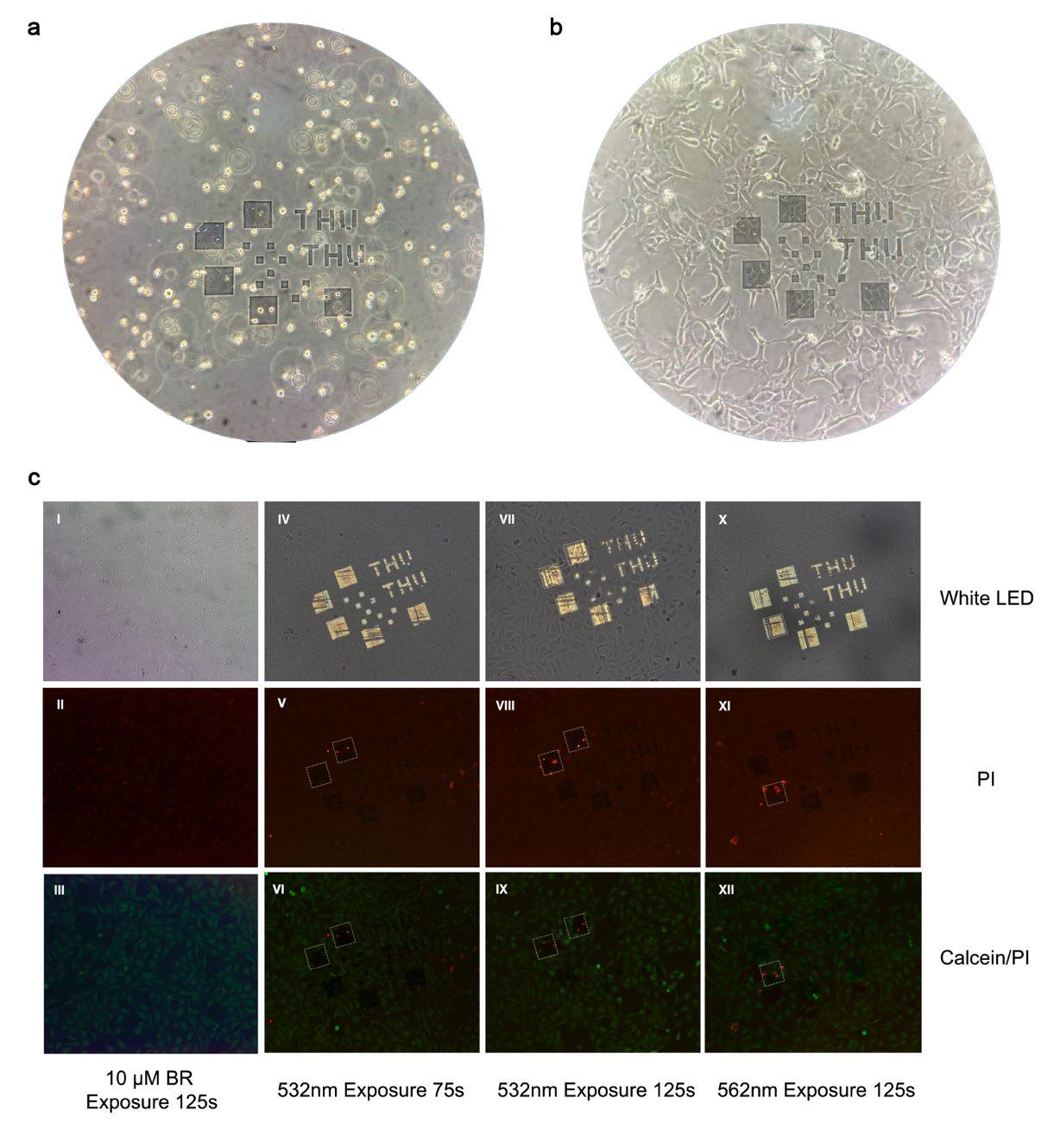


Figure S5: ***In vitro* culture of U2OS human osteosarcoma cells on an Au-TiO₂ optical metasurface.** **(a)** U2OS cells cultured on the metasurface before adhesion, showing unattached cells. **(b)** U2OS cells after 48 hours of cultivation on the metasurface at 37°C, showing attached cells.
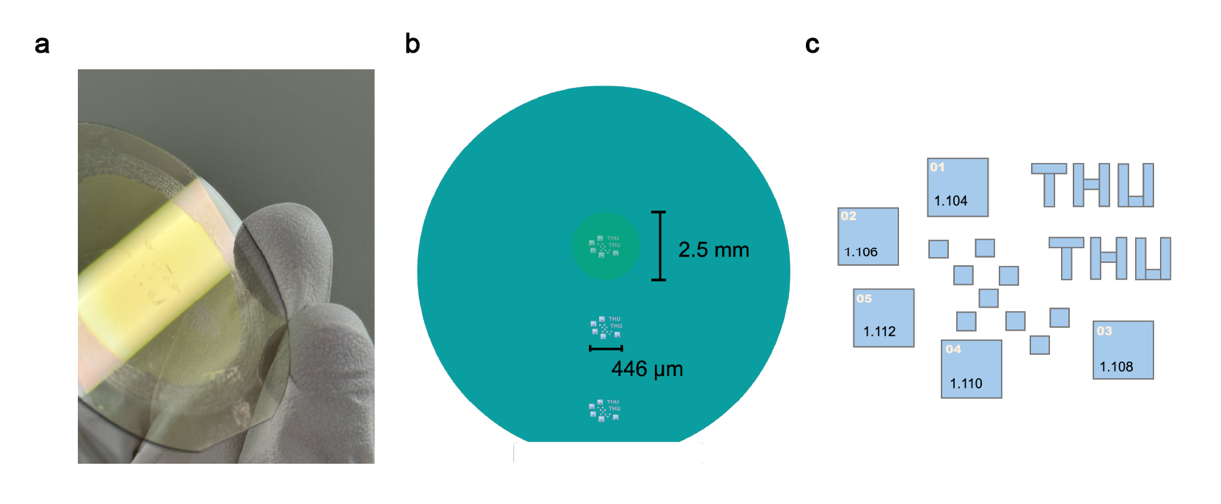


Figure S6: **Post-fabrication characterization of geometric parameter distribution and substrate morphology.** **(a)** Photograph showing the overall appearance of the SiO_2_ substrate used for metasurface fabrication. **(b)** Schematic representation of the patterned regions distributed on the circular SiO_2_ wafer. The beam spot used in the U2OS cell-killing experiment is expanded to a diameter of 2.5 mm, as indicated. **(c)** Detailed distribution of the processed surfaces with different scaling parameters ($S$) on the wafer. The black digits represent the scaling factor $S$, with $\theta$ set to 0.26 rad. The THU pattern corresponds to the region reflecting green light, as shown in Fig. 3d, g while the nine small rectangles in the center are designated for testing the electron beam exposure dose.


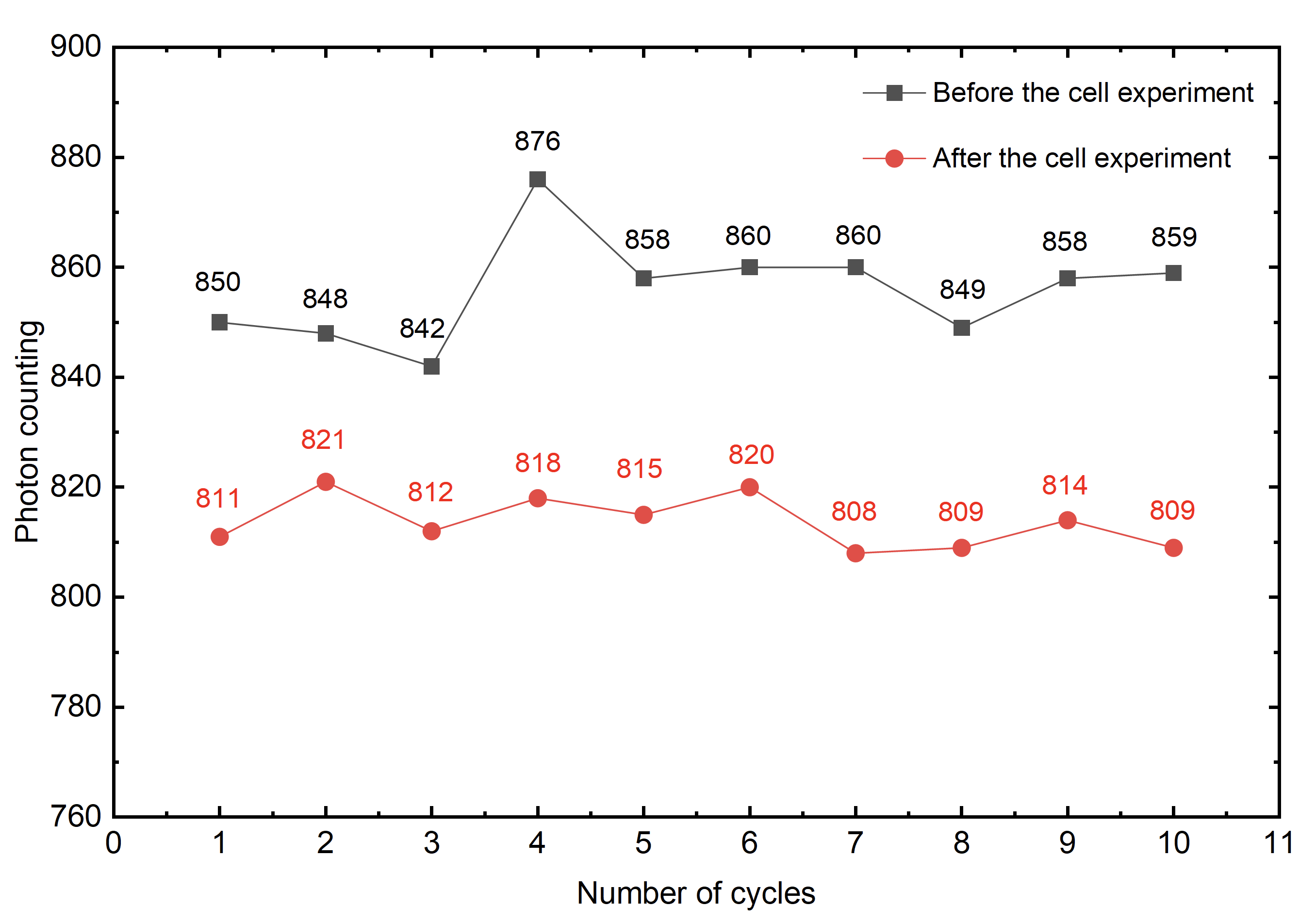


Figure S7: **Cycling stability and the effect of cell-handling on the ^1^O_2_ phosphorescence signal.** Photon counts recorded by the single-photon detector (1270-nm phosphorescence) as a function of cycle number under continuous 532-nm illumination. Each cycle consists of adding O_2_-saturated deionized water, illuminating the metasurface for 10 min through an objective of high numerical aperture, collecting the phosphorescence signal, followed by rinsing and drying the metasurface with a gentle N_2_ blow before the next run. The pristine metasurface (before the cell experiment, black squares) shows stable photon counts over 10 consecutive cycles, indicating good reusability under repeated aqueous operation. After one cell experiment (red circles), the photon counts decrease under otherwise identical conditions, which is attributed to mechanical damage (visible scratches) introduced during cell handling.

Supplementary Table 1： Effect of particle size on maximum wavelength of absorbance and molar extinction coefficient of gold nanoparticles^3^.

| Diameter  (nm) | Peak Wavelength  (nm) | Molar Extinction Coefficient  (M^-1^cm^-1^) |
| --- | --- | --- |
| 5 | 515-520 | 1.10×10^7^ |
| 10 | 515-520 | 1.01×10^8^ |
| 15 | 520 | 3.67×10^8^ |
| 20 | 524 | 9.21×10^8^ |
| 30 | 526 | 3.36×10^9^ |
| 40 | 530 | 8.42×10^9^ |
| 50 | 535 | 1.72×10^10^ |
| 60 | 540 | 3.07×10^10^ |
| 80 | 553 | 7.70×10^10^ |
| 100 | 572 | | 1.57×10^11^ | | --- | |

**Supplementary Text 1**

**Determination of the critical-coupling angle via TCMT**

To rigorously identify the critical-coupling operating point of the q-BIC metasurface, we employ temporal coupled-mode theory (TCMT)^4^, in which the total quality factor satisfies:

$$\frac{1}{Q_{tot}}=\frac{1}{Q_{rad}}+\frac{1}{Q_{abs}}$$

where $Q_{rad}$ describes radiative leakage and $Q_{abs}$ accounts for intrinsic (absorptive) loss. Under the critical-coupling condition, the radiative and intrinsic loss rates are matched, i.e.,

$$Q_{rad}\approx Q_{abs}$$

which corresponds to maximized absorptance at a given excitation wavelength.

**Estimation of Q_abs_ from an effective refractive index**

In our platform, the intrinsic loss is dominated by absorption in the ultrathin Au layer and is approximately fixed for a given material stack. We estimate $Q_{abs}$ from the effective refractive index of the integrated multilayer system. Writing

$$n_{eff}=n_{real}+in_{imag}$$

the absorptive quality factor is approximated as:

$$Q_{abs}=\frac{n_{real}}{n_{imag}}$$

We estimate $n_{eff}$ using a thickness-weighted average over the constituent layers:

$$n_{eff}=\frac{n_{w}t_{w}+n_{g}t_{g}+\left(n_{t}t_{t}+n_{s}t_{s}\right)\eta}{t}$$

Here, $n_{w}$ and $t_{w}$ are the complex refractive index and thickness of the water layer; $n_{g}$and $t_{g}$ are the complex refractive index and thickness of the Au layer; $n_{t}$ and $t_{t}$ are the complex refractive index and thickness of the TiO_2_ layer; $n_{s}$ and $t_{s}$ are the complex refractive index and thickness of the SiO_2_ layer; $\eta$ is the filling factor (duty cycle) of the Au and TiO_2_ regions; and $t$ is the total thickness of the multilayer stack. The complex refractive indices used in this estimation are taken from the refractive-index database refractiveindex.info. Using the parameters of our structure, we obtain:

$$Q_{abs}=\frac{n_{real}}{n_{imag}}=1016$$

**Extraction of Q_rad_ and determination of the critical angle**

The radiative quality factor $Q_{rad}$ is controlled by symmetry breaking of the q-BIC mode and can be tuned by the relative rotation angle $\theta$ between neighboring meta-atoms. We define the asymmetry parameter as $\alpha=\sin\left(\theta\right)$. Eigenmode simulations of the integrated system (without the 7-nm Au film) show that $Q_{rad}$ follows a power-law dependence on $\alpha$ (see Fig. 1c in the main text):

$$Q_{rad}=k\alpha^{m}$$

where the fitted parameters $k$ and $m$ are given in Fig. 1c. Combining the TCMT critical-coupling condition ($Q_{rad}=Q_{abs}$) with the fitted relation above, the critical-coupling point is reached when $Q_{rad}\left(\theta\right)=Q_{abs}$. Solving for $\theta$ yields:

$$\theta=0.259\approx0.26 rad$$

which is the operating point highlighted in Fig. 1e of the main text. This prediction is further validated by directly simulating the absorptance at $\lambda=532$nm as a function of $\theta$, where the absorptance reaches its maximum near $\theta\approx0.26$rad (Fig. 1f in the main text), consistent with the TCMT-based loss-matching analysis.

**Supplementary Text 2**

**Mechanism of Enhanced Photon Absorption in Quasi-BIC Metasurfaces**

The quasi-bound state in the continuum (q-BIC) metasurface presents a revolutionary approach to photon management, achieving exceptional photon absorption efficiency through meticulously engineered photonic resonances. Its enhancement mechanism can be attributed to symmetry-breaking photonic resonance engineering, which localizes optical energy within subwavelength dimensions and significantly enhances light-metal interactions. This enables a decoupling of absorption efficiency from metal loading. Unlike conventional optical systems, which rely on increasing the optical path length of materials to enhance absorption as dictated by the Beer-Lambert law, the metasurface fundamentally overcomes this limitation. Bloch-engineered photonic resonances facilitate multiple reflections and constructive interference in a nanoscale framework, eliminating the need for increased material thickness while maintaining exceptional absorption rates. To compare the photon absorption capabilities of the metasurface with those of gold nanoparticles, we employ the Beer-Lambert law under identical absorption conditions：

$$\log\left(\frac{1}{T}\right)=A=\varepsilon\cdot c\cdot l$$

where $A$ is the dimensionless absorbance, $T$ denotes transmittance ($T=I/I_{0}$ with $I$ and $I_{0}$ representing transmitted and incident light intensities respectively), $\varepsilon$ is the molar attenuation coefficient (typically in M^-1^cm^-1^) dependent on both the absorbing material's properties and the incident light wavelength $\lambda$, $c$ signifies the molar concentration of the absorbing species (M), and $l$ represents the optical path length in centimeters (cm).

(i) The designed metasurface attenuates 45.4% of incident laser energy. To determine the equivalent Au volume per unit area $S$ (laser-irradiated area) required for this attenuation level, we analyze its unit cell structure: Two elliptical gold films per unit cell with individual area $s_{1}=\pi\cdot A\cdot B$, where $A$ and $B$ denote semi-major and semi-minor axes. Given the unit cell dimensions $s_{2}=P_{x}\times P_{y}$, the Au area fraction is $\eta=2\pi AB/P_{x}P_{y}$. With a Au film thickness of $d=7$nm, the equivalent Au volume per unit area is calculated as:

$$V_{1} =d\cdot\eta\cdot S=(7\times10^{-9})\times0.4046\times S=2.832\times10^{-7}\cdot S\text{ }[\text{cm}^{3}]$$

(ii) To determine the required Au volume per unit area $S$ (laser-irradiated area) for nanoparticle-based attenuation at this level, we employ 40-nm-diameter gold nanoparticles as the target material. These nanoparticles exhibit an absorption peak at 530 nm wavelength with a molar extinction coefficient of $\varepsilon=8.42\times10^{9}$ M⁻¹·cm⁻¹ (Supplementary Table 1). The volumetric gold requirement is then calculated as derived from the Beer-Lambert law.

$$\log\left(\frac{1}{T}\right)=\log\left(\frac{1}{1-0.454}\right)=0.263=\varepsilon\cdot c\cdot l=\varepsilon\cdot c\cdot\frac{V_{0}}{S}=\varepsilon\cdot\frac{n}{S}=\varepsilon\cdot\frac{N}{N_{mol}\cdot S}$$

The irradiated solution volume $V_{0}$ contains Au nanoparticles with molar quantity $n=c\cdot V_{0}$ (where $c$ denotes molar concentration), and the nanoparticle count $N$ is given by $N=n\cdot N_{A}$ with $N_{A}$ representing Avogadro's constant ($6.022\times10^{23}$mol^-1^). Consequently, the required Au volume $V_{2}$ for attenuating 45.4% of incident laser energy via nanoparticle absorption is derived from Equation:

$$V_{2}=\frac{4}{3}\pi r^{3}\cdot N=\frac{4}{3}\pi\times(20\text{ nm})^{3}\times0.263\times\frac{N_{mol}\cdot S}{\varepsilon}=6.301\times10^{-4}\cdot S\text{ }[\text{cm}^{3}]$$

Comparative analysis of Equations and reveals that the metasurface approach demonstrates superior performance. The required gold loading is merely ~0.045% of that for nanoparticle-based systems. This substantial reduction in gold mass absorbing equivalent laser energy will significantly elevate electron temperatures ($T_{e}$) and photoactivate charge carriers.

**Supplementary Text 3**

**Calculation of the Heterojunction Current in the Au-TiO_2_ System**

In the thermionic emission theory, the magnitude of the heterojunction current is related to the electron temperature $T_{e}$. Therefore, we need to use the two-temperature model to calculate the electron temperature that the fabricated Au-TiO_2_ heterojunction metasurface can reach under continuous laser irradiation, and then calculate the magnitude of the heterojunction current based on the electron temperature $T_{e}$. According to the two-temperature model^5^:

where $t$ represents time, the electron temperature is denoted as $T_{e}$, and the lattice temperature is denoted as $T_{l}$, both expressed in units of Kelvin (K). The term $C_{e}\left(T_{e}\right)$ corresponds to the electron heat capacity per unit volume, while $C_{l}\left(T_{l}\right)$ represents the lattice heat capacity per unit volume, both expressed in units of J/m^3^/K. The function $S\left(x,t\right)$ represents the heat source input per unit volume, which, in our case, corresponds to the laser input. We assume that $S\left(x,t\right)$ does not vary with time, and its units are W$\cdot$m^-3^. The electron-phonon coupling coefficient, $G_{e}\left(T_{e}\right)$, is expressed in the units of W$\cdot$m^-3^$\cdot$K^-1^ and characterizes the rate of energy exchange between electrons and the lattice.

The spatial derivative terms in the equations represent the transfer of thermal energy from electrons (or the lattice) to surrounding electrons (or the lattice) in adjacent spatial locations. When the fabricated metasurface reaches thermal equilibrium under laser illumination, the terms $dT_{e}/dt$ and $dT_{l}/dt$ become zero. Under these conditions, we obtain the following equations:

$$G_{e}V\left(T_{e}-T_{l}\right)=S_{0}$$

This equation provides the relationship among the laser energy input, the effective volume of the metal, and the electron-lattice temperature difference under steady-state conditions, where $V$ represents the volume of the Au irradiated by the laser beam, and $S_{0}$ denotes the input laser energy, which is maintained at 25 mW in the experiment. The electron-phonon coupling coefficient of Au is taken as $G_{e}=2.2\times10^{16}$W$\cdot$m^-3^$\cdot$K^-1^. This equation indicates that, for the same laser energy input, a smaller volume of Au results in a larger temperature difference between the electrons and the lattice. This phenomenon highlights the contribution of BIC photonic engineering.

The comparison between the photocurrent generation capability in the metasurface-based Au-TiO_2_ heterojunctions and conventional nanoparticle-based configurations is necessary to evaluate the efficiency improvements enabled by the quasi-BIC design and quantify the advantages of spatially localized charge separation inherent in the metasurface architecture.

(i) Calculation of Photocurrent Generation in Au-TiO_2_ Heterojunctions Metasurface

In the experiment, the volume of the laser-irradiated region, after being focused by the objective lens, is calculated as:

$$V=\eta\pi r^{2}d$$

where $\eta$ and $d$ are introduced in Supplementary Text 1, representing the Au filling ratio of the metasurface and the thickness of the gold thin layer, respectively.$r$ denotes the radius of the laser beam focused on the sample surface by the objective lens. Additionally, the effective volume is considered to be 13.49% of the actual volume (as shown in Fig. S2). Therefore, the effective volume is given by:

$$V_{eff}=13.49\%\cdot V$$

The difference between the electron temperature and the lattice temperature is expressed as:

$$\Delta T=T_{e}-T_{l}=\frac{S_{0}}{G_{e}V_{eff}}$$

where $T_{e}$ and $T_{l}$ are the electron and lattice temperatures, respectively. Au exhibits a relatively low electron-phonon coupling coefficient compared to other metals. Additionally, the metasurface is immersed in water, which has a high heat capacity. As a result, the lattice temperature is assumed to be equal to the ambient temperature, $T_{l}\approx298.15$K. Using this assumption, the electron temperature is calculated as $T_{e}\approx308.9$K. In the thermionic emission model, the current density $J$ is expressed as^6^:

where $J$ represents the current density generated by thermally excited carriers, with the units of A/cm^2^. Here, $q$ is the elementary charge, is the Schottky barrier height between Au and TiO_2_, $k_{B}$ is the Boltzmann constant, $h$ is the Planck constant, and $m_{n}$ is the effective mass of the carriers. For metals, $m_{n}$ is taken as the electron mass. Typically, the Schottky barrier height is approximately eV. The total photocurrent $I$ in the heterojunction can be calculated based on the illuminated area as:

$$I=J\cdot\pi r^{2}\cdot\eta$$

For a 25 mW laser beam focused on the Au-TiO_2_ heterojunction metasurface with a focal spot radius of $r=10$µm, the thermally excited current in the heterojunction increases by approximately 2.6 pA.

(ii) Calculation of Photocurrent in Au-TiO_2_ Spherical Heterojunction Nanoparticles

The electron temperature generated by the absorption of an equivalent amount of laser energy is estimated for 40 nm diameter Au nanoparticles (coated with a TiO_2_ outer layer). The irradiated volume of the gold nanoparticles is given by:

$$V_{particels}=\frac{4}{3}\pi R^{3}\cdot N$$

where $R$ is the radius of the Au nanoparticles, and $N$ represents the number of heterojunction nanoparticles exposed to the laser beam. Using the same calculation method as described above, the current density $J_{particles}$ for the heterojunction can be determined. The total photocurrent generated across all heterojunction interfaces is expressed as:

$$I_{particles}=J_{particles}\cdot4\pi R^{2}\cdot N$$

where $4\pi R^{2}\cdot N$ represents the total contact area between all Au nanoparticles and their TiO_2_ coatings. For a 25 mW laser beam focused on Au-TiO_2_ spherical heterojunction nanoparticles within a beam radius of $r=10$µm, the thermally excited current in the heterojunction increases by approximately 0.42 pA.

**Supplementary Text 4**

**Fabrication method of Au-TiO_2_ Metasurfaces**

The fabrication method of the Au-TiO₂ heterojunction metasurface is presented as follows.

(i) Substrate cleaning. A 2-inch BF33 float glass substrate is selected. The substrate is immersed in acetone and ethanol sequentially, and cleaned for 10 minutes under an ultrasonic power of 200 W. It is then rinsed with deionized water, dried with a nitrogen gun, and placed in an oven at 120°C for 30 minutes.

(ii) Spin coating. 1 mL of 950PMMA A4 resist (Kayaku Advanced Materials, Inc.) is dispensed onto the clean substrate. The rotational speed and duration of the EZ4 spin coater (Henan Mingguan Scientific Instruments Co., Ltd.) are set to 3000 revolutions per minute and 45 seconds, respectively. After spin coating, the substrate is baked on a hot plate at 180 °C for 90 seconds. Following cooling, a 40-nm diameter gold nanoparticle solution (KYKY Technology Co., Ltd.) is applied to the sample for focusing.

(iii) Pattern formation. The spin-coated sample is placed in the process chamber of the NB5 electron beam lithography machine (Nanobeam Ltd) under a vacuum of less than 2.7e-8 Torr. The electron gun acceleration voltage is set to 80 kV, the current to 0.8 nA, and the pattern dose to 8 C$\cdot$m^-2^. The exposed sample is developed in MIBK/IPA 1:3 (Kayaku Advanced Materials, Inc.) for 60 seconds and fixed in isopropyl alcohol for 60 seconds. It is finally dried with a nitrogen gun.

(iv) Film deposition and lift-off. The patterned sample is placed in the process chamber of the PVD75 electron beam evaporation coating machine (The Kurt J. Lesker Company) under a vacuum of less than 1e-7 Torr. The deposition rate is controlled at 1 Å$\cdot$s^-1^ using the PVD75 standard automated program, depositing a 100 nm TiO₂ film followed by a 7 nm Au film. The completed sample is immersed in acetone for 48 hours, dissolving the resist and thereby detaching the overlying TiO₂ and Au films. This process yields regular TiO₂-Au dielectric elliptical cylinders.

**Supplementary Text 5**

**Calculation of Singlet Oxygen Concentration and Quantum Yield**

We calculate the number, concentration, and quantum yield of singlet oxygen molecules generated by the metasurface through a comparison with the photosensitizer Rose Bengal. In the time-correlated single-photon counting (TCSPC) experiment for singlet oxygen phosphorescence, we observe that the photon count of singlet oxygen phosphorescence generated by the fabricated optical metasurface is at the same level as that of a 10 μM Rose Bengal deuterated water solution with a path length of 1 cm.

To estimate the number of singlet oxygen molecules generated by the 10 μM Rose Bengal deuterated water solution, we consider the following parameters: the concentration of the Rose Bengal solution is $c=10\text{ }$μM, the quantum yield is $\Phi_{\Delta}=0.76$^7,8^, the laser power is $P=25\text{ }$mW, and the optical path length is $l=1\text{ }$cm. The experimentally measured fraction of laser energy absorbed by the solution is 84.2%. The number of photons absorbed by the photosensitizer per unit time is calculated as:

$$N=0.842\frac{P}{E_{ph}}=0.842\frac{P\lambda}{hc}=5.63\times10^{16}\text{photons}\cdot\text{s}^{-1}$$

where $E_{ph}$ is the energy of a single photon, $\lambda$ is the laser wavelength, $h$ is Planck's constant, and $c$ is the speed of light. Using the definition of quantum yield, the number of singlet oxygen molecules generated per unit time is estimated as:

$$N_{1}=\Phi_{\Delta}\cdot N=4.28\times10^{16}\text{molecules}\cdot\text{s}^{-1}$$

From the photon counting experiment, we determine that the number of singlet oxygen molecules generated by the metasurface $N_{2}$ is at the same level as $N_{1}$. Therefore, we have:

$$N_{1}=N_{2}$$

However, in the case of the Rose Bengal solution, these singlet oxygen molecules are produced within a 1 cm thick solution layer and are distributed around the irradiated optical region. This fundamentally differs from the generation mechanism of singlet oxygen by the metasurface, where the singlet oxygen molecules are distributed in the region above the metasurface, diffusing over a certain distance defined as the diffusion length ($r$).

(i) First, we calculate the volume $V_{\text{RB}}$ of the region where singlet oxygen molecules are distributed in the Rose Bengal solution (Fig. S4):

$$V_{\text{RB}}=\frac{2}{3}\pi\cdot\rho^{2}\cdot h$$

where this region can be approximated as the combined volume of two cones. Each cone has a height $h=0.5$cm and a base radius $\rho\approx0.5$ mm, based on experimental measurements. Due to the relatively large dimensions of the cones compared to the diffusion length of singlet oxygen, the effects resulting from the diffusion length are neglected in this calculation.

(ii) Next, the volume of the region where singlet oxygen molecules are distributed above the metasurface, $V_{\text{meta}}$, is calculated as:

$$V_{\text{meta}}=\left(S_{\text{meta}}\right)^{2}\cdot r=\left(S_{\text{meta}}\right)^{2}\cdot\sqrt{6D\tau}$$

where $S_{\text{meta}}$ denotes the size of the patterned metasurface area, $r$ represents the diffusion length of singlet oxygen molecules, $D=1.6\times10^{-5}$cm^2^/s is the diffusion coefficient of singlet oxygen in deuterated water^9^, and $\tau=64$µs is the singlet oxygen lifetime in deuterated water^8^.

Based on the calculated volumes, the steady-state concentration of singlet oxygen generated by the two methods can be compared using the formula^10,11^:

$$\left[\text{O}_{2}\right]=R_{\text{O2}}\cdot\tau=\frac{N_{\text{O2}}}{N_{A}\cdot V}\cdot\tau$$

where $N_{\text{O2}}$ can take the values $N_{1}$ or $N_{2}$, $V$can take the values $V_{\text{RB}}$ or $V_{\text{meta}}$, and $N_{A}$is Avogadro’s constant, $R_{\text{O2}}$ is the generation rate of singlet oxygen. The singlet oxygen concentration generated by the Rose Bengal solution is calculated to be 0.87 µM, whereas the localized singlet oxygen concentration generated near the metasurface reaches 1.12 M. This significant enhancement by 6 orders of magnitude is attributed to the superior electron transfer capability and oxygen molecule adsorption capacity of the Au-TiO_2_ heterostructured metasurface.

Furthermore, the quantum yield of singlet oxygen generated by the metasurface, $\Phi_{\Delta}^{\text{meta}}$, can be determined using the following expression:

$$\Phi_{\Delta}^{\text{meta}}=\Phi_{\Delta}^{\text{RB}}\times\frac{I_{\text{meta}}}{I_{\text{RB}}}\times\frac{A_{\text{RB}}}{A_{\text{meta}}}$$

where $\Phi_{\Delta}^{\text{RB}}$ is the quantum yield of singlet oxygen in the Rose Bengal solution,$I_{\text{meta}}$ and $I_{\text{RB}}$ are the incident light intensities for the metasurface and the Rose Bengal solution, respectively, and $A_{\text{meta}}$ and $A_{\text{RB}}$ are the absorption coefficients for the two systems. The absorption coefficient of the metasurface is calculated to be 1.42, and the quantum yield exceeding 1 indicates that the metasurface enables multiple electron excitations per photon, producing more singlet oxygen molecules than incident photons. This result further validates the exceptional performance of the metasurface in singlet oxygen generation.

**Supplementary Text 6**

**Protocol for the Preparation of Singlet Oxygen Sensor Green (SOSG) Solution**

A notable limitation of the SOSG reagent is its potential instability, self-activation, and even singlet oxygen generation when exposed to shorter-wavelength light (UVA, blue, and green) in the absence of any photosensitizer (PS)^12^. To validate the singlet oxygen generation capability of the metasurface, a non-patterned area combined with a 10 μM BR solution is used as a blank control. The average value is calculated from four samplings to reduce noise. For the preparation of the SOSG solution, 100 μg of the reagent is dissolved in 33 μL of methanol to produce a methanol stock solution with a concentration of approximately 5 mM. This stock solution is then diluted to 10 μM using deionized water and applied to the optical metasurface, which is covered by a water layer. A laser beam is focused on the patterned metasurface and a 1 cm water layer containing the 10 μM BR solution to obtain the curve shown in Fig. 5a in the main text.

**References**

1. Manrique-Bedoya, S. *et al.* Multiphysics Modeling of Plasmonic Photothermal Heating Effects in Gold Nanoparticles and Nanoparticle Arrays. *J. Phys. Chem. C* **124**, 17172–17182 (2020).

2. Baffou, G. & Quidant, R. Thermo‐plasmonics: using metallic nanostructures as nano‐sources of heat. *Laser Photonics Rev.* **7**, 171–187 (2013).

3. Dolinnyi, A. I. Extinction coefficients of gold nanoparticles and their dimers. Dependence of optical factor on particle size. *Colloid J.* **79**, 611–620 (2017).

4. Jin, R. *et al.* Toroidal Dipole BIC-Driven Highly Robust Perfect Absorption with a Graphene-Loaded Metasurface. *Nano Lett.* **23**, 9105–9113 (2023).

5. Taghinejad, M. *et al.* Determining hot-carrier transport dynamics from terahertz emission. *Science* **382**, 299–305 (2023).

6. Hu, C. PN and metal-semiconductor junctions. *UC Berkeley EE Cl. Note* 89–156 (2009).

7. Szewczyk, G. & Mokrzyński, K. Concentration-Dependent Photoproduction of Singlet Oxygen by Common Photosensitizers. *Molecules* **30**, 1130 (2025).

8. Williams, G. O. S., Euser, T. G., Russell, P. St. J., MacRobert, A. J. & Jones, A. C. Highly Sensitive Luminescence Detection of Photosensitized Singlet Oxygen within Photonic Crystal Fibers. *ChemPhotoChem* **2**, 616–621 (2018).

9. Han, P. & Bartels, D. M. Temperature Dependence of Oxygen Diffusion in H_2_O and D_2_O. *J. Phys. Chem.* **100**, 5597–5602 (1996).

10. Ossola, R., Jönsson, O. M., Moor, K. & McNeill, K. Singlet Oxygen Quantum Yields in Environmental Waters. *Chem. Rev.* **121**, 4100–4146 (2021).

11. Partanen, S. B., Apell, J. N., Lin, J. & McNeill, K. Factors affecting the mixed-layer concentrations of singlet oxygen in sunlit lakes. *Environ. Sci. Process. Impacts* **23**, 1130–1145 (2021).

12. Flors, C. Imaging the production of singlet oxygen in vivo using a new fluorescent sensor, Singlet Oxygen Sensor Green(R). *J. Exp. Bot.* **57**, 1725–1734 (2006).
